# Multimodal, Multiscale Insights into Hippocampal Seizures Enabled by Transparent, Graphene-Based Microelectrode Arrays

**DOI:** 10.1523/ENEURO.0386-21.2022

**Published:** 2022-05-09

**Authors:** Patrick J. Mulcahey, Yuzhang Chen, Nicolette Driscoll, Brendan B. Murphy, Olivia O. Dickens, A. T. Charlie Johnson, Flavia Vitale, Hajime Takano

**Affiliations:** 1 Division of Neurology, Department of Pediatrics, The Children’s Hospital of Philadelphia, Philadelphia, PA 19104; 2 Department of Bioengineering, University of Pennsylvania, Philadelphia, PA 19104; 3 Department of Neuroscience, Perelman School of Medicine, University of Pennsylvania, Philadelphia, PA 19104; 4 Center for Neuroengineering and Therapeutics, University of Pennsylvania, Philadelphia, PA 19104; 5 Center for Neurotrauma, Neurodegeneration, and Restoration, Corporal Michael J. Crescenz Veterans Affairs Medical Center, Philadelphia, PA 19104; 6 Graduate Group in Biochemistry and Molecular Biophysics, University of Pennsylvania, Philadelphia, PA 19104; 7 Department of Physics and Astronomy, University of Pennsylvania, Philadelphia, PA 19104; 8 Department of Neurology, Perelman School of Medicine, University of Pennsylvania, Philadelphia, PA 19104; 9 Department of Physical Medicine and Rehabilitation, University of Pennsylvania, Philadelphia, PA 19104

**Keywords:** calcium imaging, epilepsy, hippocampus, microelectrode

## Abstract

Hippocampal seizures are a defining feature of mesial temporal lobe epilepsy (MTLE). Area CA1 of the hippocampus is commonly implicated in the generation of seizures, which may occur because of the activity of endogenous cell populations or of inputs from other regions within the hippocampal formation. Simultaneously observing activity at the cellular and network scales *in vivo* remains challenging. Here, we present a novel technology for simultaneous electrophysiology and multicellular calcium imaging of CA1 pyramidal cells (PCs) in mice enabled by a transparent graphene-based microelectrode array (Gr MEA). We examine PC firing at seizure onset, oscillatory coupling, and the dynamics of the seizure traveling wave as seizures evolve. Finally, we couple features derived from both modalities to predict the speed of the traveling wave using bootstrap aggregated regression trees. Analysis of the most important features in the regression trees suggests a transition among states in the evolution of hippocampal seizures.

## Visual Abstract

**Figure:**
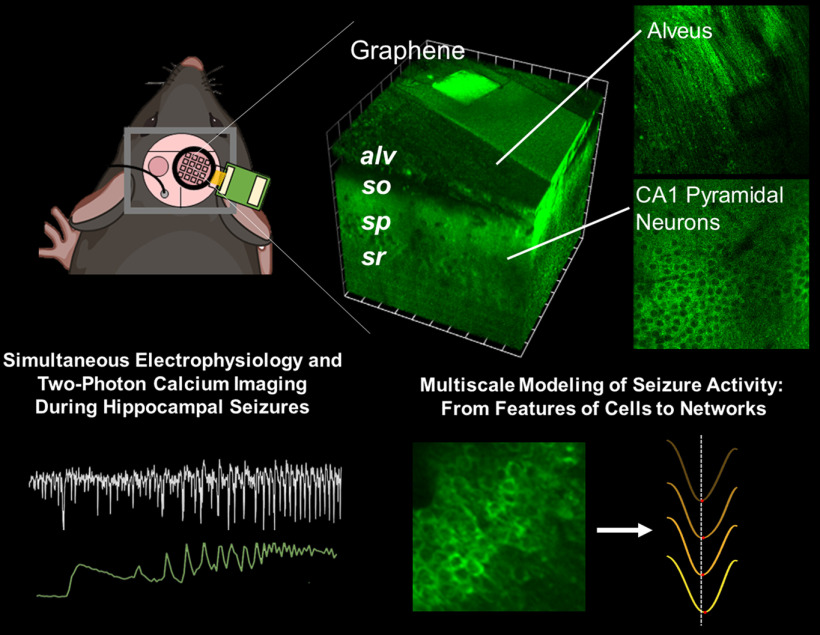

## Significance Statement

There is a pressing need to develop novel technological strategies to integrate modalities toward greater mechanistic understanding of neuronal activities and brain computer interfacing applications. Our study provides an important advance by introducing a cannula imaging window/transparent electrode assembly to perform simultaneous multimodal measurements within mouse hippocampus. Performing and making sense of simultaneous measurements from the scale of single cells to a network level remains a substantial technical and conceptual challenge. Coupling measurements made by our methodology with an interpretable machine learning algorithm allows us to overcome this problem and posit a role of inhibition during hippocampal seizures.

## Introduction

Seizures are the hallmark feature of epilepsy, a neurologic disorder that affects 2.2 million people in the United States alone (England et al., 2012). Temporal lobe epilepsy (TLE) is the most common form of epilepsy, accounting for ∼60% of all epilepsy patients. Approximately 80% of TLE patients are diagnosed with mesial TLE (MTLE), an epilepsy subtype that implicates the hippocampus and the surrounding brain regions in the generation of often drug-refractory seizures (Lévesque et al., 2018). Despite the high clinical prevalence of MTLE, the mechanisms of seizure initiation and evolution in the hippocampus remain poorly understood.

Of the subfields of the hippocampus, CA1 has historically been regarded as an important site for seizure initiation and evolution. In the broader context of the hippocampal-entorhinal system, CA1 often succumbs to seizure-promoting inputs from CA3 and the entorhinal cortex (Dulla et al., 2018). Early work in *in vitro* slice models of seizure-like events implicated the CA3-CA1 axis in the generation of epileptiform activity. In these models, slices are bathed in solutions with an ionic imbalance (low Mg^2+^, Cl^–^, high K^+^) or pharmacological convulsants [picrotoxin, 4aminopyridine (4-AP)], resulting in spontaneous discharges that originate in CA3, propagate along the highly recurrent, excitatory Schaffer collaterals, and induce a depolarization associated with a burst of action potentials in the cell populations of CA1 (Dulla et al., 2018). Moreover, voltage sensitive dye imaging studies in rodent epileptic hippocampal slices demonstrated a substantial upregulation of the excitatory input of the temporoammonic pathway from entorhinal cortex layer III to CA1 (Ang et al., 2006). At the cellular scale in area CA1, there has been a substantial focus on pathologic activity inducing the development of “burster” neuron populations in epilepsy (Jensen and Yaari, 1997; Chen et al., 2011; Takano et al., 2012). In epileptic CA1, a fraction of the pyramidal cell (PC) population converts from regular firing modes (the predominant spiking mode in normal conditions) to a bursting mode. In the bursting mode, cells may fire action potentials spontaneously or in dense clusters in response to threshold stimulation. Importantly, bursting PC neurons can initiate network bursts in CA1 and are thought to play a significant role in the initiation of epileptiform events.

These insights, predominately derived from *in vitro* studies of the hippocampus, emphasize the multiscale nature of epileptic activity in CA1. Seizures may either be driven both by the endogenous populations of excitable cells in CA1 or by network level interactions with the structures that innervate it. Relating these scales of activity *in vivo* in the hippocampus remains both a significant technical and conceptual challenge. Electrophysiology is the gold standard technique for recording seizures, but electroencephalography (EEG) lacks the fine spatial resolution required to resolve the activity of CA1 microcircuits (Farrell et al., 2019). Single or multishank high-density laminar probes can resolve the activity of microcircuits but cannot resolve the behavior of individual cells during seizure, as local hypersynchrony and changes in action potential waveform shape following seizure recruitment render spike sorting intractable (Merricks et al., 2015). Cellular imaging techniques, such as two-photon multicellular calcium imaging, can resolve the behavior of genetically defined neural populations with single cell resolution, although they sacrifice the temporal resolution of electrophysiology (Yaksi and Friedrich, 2006; Takano and Coulter, 2012). Single-scale observations of the hippocampus during seizure inherently limit our conceptual understanding of how these scales interact. For example, in cortical epilepsy, multiscale, nonlinear models of seizure activity were developed only after simultaneous macroscale electrocorticography and Utah microarray recordings were introduced clinically (Eissa et al., 2017; Schevon et al., 2019). To overcome the technical limitations of single-modality measurements, transparent microelectrode arrays (MEAs) have emerged in recent years as a family of novel neuro-technologies that enable simultaneous electrophysiology and calcium imaging (Kuzum et al., 2014; Park et al., 2014; Driscoll et al., 2021; Liu et al., 2021). Previously, low-magnification calcium imaging combined with a transparent MEA recordings in 4-aminopyridine -induced cortical seizures revealed the evolution of hidden modes throughout seizure recruitment, evolution, and termination (Driscoll et al., 2021). In particular, this study emphasized the unique contribution of widefield calcium imaging, whose slow propagation and evolution resembled the spread of a core seizure-recruited territory, or “ictal core,” to electrophysiological recording of seizures.

To address these challenges, we propose a novel methodology for simultaneous electrophysiological recording and two-photon multicellular calcium imaging of CA1 PCs during acutely-induced seizures *in vivo*. Our strategy is enabled by fabricating an implantable imaging window integrated with an optically transparent MEA based on graphene. This methodology allows us to image the activity of hundreds of neurons within a 300 × 300 μm^2^ area while recording electrical signals from an area covering 1.55 × 1.55 mm^2^. Bridging modalities and scales, we examine the recruitment of PCs at seizure onset, the oscillatory organization of neural ensembles during the established seizure, and the spatiotemporal characteristics of the low-frequency seizure traveling wave throughout seizure evolution. Finally, we combine features derived from multicellular calcium imaging and single site electrophysiology to predict the speed of the seizure traveling wave using bootstrap aggregated regression trees. By examining the time evolution of the most important features in the regression trees, we note substantial changes in feature values co-incident with changes in the speed of the traveling wave, suggesting a dynamic transition through states as seizures evolve and terminate in CA1.

## Materials and Methods

All animal use was performed in accordance and with the approval of the Children’s Hospital of Philadelphia’s Institutional Animal Care and Use Committee. A total of 11 mice of either sex were used in this study. Four mice expressing genetically encoded calcium indicators were implanted with transparent MEAs, resulting in three high-quality multimodal datasets and one calcium imaging-only dataset. Seven wild-type mice were implanted with 32-channel multishank probes, resulting in seven high-quality electrophysiology-only datasets.

### Multimodal recordings with graphene-based MEAs (Gr MEAs)

Transparent, flexible MEAs based on graphene were fabricated as described previously (Driscoll et al., 2021). The impedance of the graphene channels was 1.95 ± 0.33 MΩ (mean ± SD). First, a 3-mm cover glass imaging window (Warner CS-3R-0) was affixed to a custom-made, hat-shaped steel cannula with an outer diameter of 3 mm (University of Pennsylvania, Vision Research Center Machine Shop) using Norland Optical Adhesive NOA 60 (Edmund Optics). Then, graphene devices were secured to the cover glass surface using the same adhesive. Eight- to 12-week-old mice expressing genetically encoded calcium indicators (*n* = 4, C57BL6-Thy1-GCaMP6s and C57BL6-Thy1-jRGECO1a, The Jackson Laboratory) were anesthetized with isoflurane anesthesia (1–2%) in oxygen. A stainless-steel screw attached to silver wire was implanted bilaterally in the skull above the right cerebellum, and a metal headplate (Narishige USA) was fixed to the skull with dental acrylic (Lang Dental). In the left hemisphere, a 2.0-mm diameter craniotomy was drilled, and the dura was removed (anteroposterior: ∼−1.0 mm, mediolateral: ∼−2.0 mm). In the right hemisphere, a 3.0-mm diameter craniotomy was drilled, and the dura was removed (anteroposterior: ∼−2.0 mm, mediolateral: ∼2.0 mm). The cortex was gently aspirated with constant application of artificial CSF (ACSF; in mm, 125 NaCl, 2.5 KCl, and 1.25 NaH_2_PO_4_) to reveal the fibers of the alveus.

Following completion of the surgery, the anesthetic was switched to ketamine/xylazine (100/10 mg/kg), and the headplate was attached to a small imaging platform (Narishige USA). This platform was mounted on a fixed stage, and imaged with a two-photon microscope (Thorlabs Begamo Scope). For imaging, the field was centered over a graphene electrode pad using a 16× objective (Nikon, 0.8 N.A., 3 mm WD). The objective was then lowered until CA1 pyramidal neurons could readily be visualized in the field. GCaMP6s was excited at 920 nm and jRGECO1a was excited at 1040 nm with a femtosecond-pulsed two photon laser (Mai Tai DeepSee, Spectra-Physics). Images were captured in a single plane at a constant acquisition rate between 5 and 10 Hz. To block ambient light, all imaging sessions were performed with the microscope enclosed in a dark chamber. The imaging data were captured in binary format and converted to TIF format on a PC, and timing of electrophysiological recording and imaging was synched by a TTL signal. The MEA was connected to a custom connector that interfaced with the Smartbox V2 (Neuronexus). For recording, the graphene electrode array, a custom connector, and the headstage (SmartLink16, NeuroNexus) were attached to a stainless-steel bar/micromanipulator ensemble. Electrophysiological data were sampled at 20 kHz via the headstage and the controller (SmartBox, NeuroNexus), and the data were saved to a PC in an Intan file format. A 5-min epoch of baseline activity was recorded and imaged before seizure induction. Seizures were induced pharmacologically by manually applying 20 μl of 4°C 15 mm 4-AP in ACSF to the cortical surface exposed by the left portal craniotomy. Hippocampal activity was recorded continuously for 2–3 h after application of 4-AP. At the end of experiments, image stacks of the area underneath the graphene device/imaging window were captured *in vivo* in the two-photon microscope.

### Multishank probe recordings

Eight- to 12-week-old C57BL/6 mice (*n* = 7, The Jackson Laboratory) were anesthetized with isoflurane anesthesia (1–2%) in oxygen. Two stainless steel screws attached to silver wires were implanted bilaterally in the skull above the cerebellum, and a metal headplate (Narishige USA) was fixed to the skull with dental acrylic (Lang Dental). In the left hemisphere, a 2.0-mm diameter craniotomy was drilled, and the dura was removed (anteroposterior: ∼−1.0 mm, mediolateral: ∼−2.0 mm). In the right hemisphere, a rectangular craniotomy was drilled, and the dura was removed (anteroposterior: ∼−2.0 mm, mediolateral: ∼2.0 mm). The anesthetic was then switched to ketamine/xylazine (100/10 mg/kg), and the headplate was attached to a small platform (Narishige USA). A 32-channel multishank probe (Neuronexus A4x8-5 mm-200–400-177-A32) sensitized with 25 mg/ml 1,1′dioctadecyl 3,3,3′,3′ (DiI; Thermo Scientific) in ethanol was targeted to the right dorsal hippocampus (anteroposterior: ∼−2.0 mm, mediolateral: ∼0.7 mm, dorsoventral: ∼−2.0 mm). Contacts for the ground and reference were made contralateral and ipsilateral to the recording site, respectively. Recordings were sampled at 20 kHz via the Neuronexus Acute Smartlink32 Headstage and Smartbox. Following 10 min of baseline recording, 20 μl of 4°C 15 mm 4-AP in ACSF (in mm, 125 NaCl, 2.5 KCl, and 1.25 NaH_2_PO_4_) was applied to the exposed cortex contralateral to the recording site, as during recordings made with the Gr MEA. Hippocampal activity was recorded continuously for 2–3 h after application of 4-AP. After completion of the recordings, mice were transcardially perfused with sucrose-rich ACSF (in mm, 75 sucrose, 87 NaCl, 2.5 KCl, 1.25 NaH_2_PO_4_, 10 glucose, 26 NaHCO_3_, 2 CaCl_2_-H_2_O, and 1 MgSO_4_) and decapitated under deep isoflurane anesthesia. Brains were removed and submerged in ice cold sucrose-rich ACSF. Coronal hippocampal slices (400 μm thick) were cut using a Vibratome VT1200S (Leica). Slices were imaged using an Olympus FV10000 confocal microscope (Olympus Corporation) with a 543-nm excitation wavelength (Melles Griot). Multishank probe locations were then co-registered with the Allen Brain Atlas using a custom-written MATLAB script (R2019b MathWorks).

### Analysis of the electrophysiological and imaging data

All data analysis was performed in MATLAB R2019b. Statistical analyses were performed in MATLAB R2019b or Prism 9 (GraphPad). To facilitate the reproducibility of this work, all custom-written MATLAB code used for analysis is available on GitHub at www.github.com/mulcaheyp. Only data obtained from graphene microelectrodes with 1000-Hz impedance between 900 kΩ and 5 MΩ were considered for analysis. Only data obtained from multishank probe channels localized to CA1 (stratum oriens, stratum pyrimidale, stratum radiatum, stratum lacunosum moleculare) were considered for analysis.

#### Calcium transient onset analyses

All calcium imaging data were registered in Suite2P. Circular regions of interest (ROIs) were manually drawn in FIJI (ImageJ, NIH) around putative CA1 PCs. To minimize the intrusion of the neuropil signal, we performed ROI shrinking by decreasing the ROI by two pixels using a custom Java macro (Wenzel et al., 2017, 2019). Fluorescence traces were extracted from the images, imported into MATLAB, normalized to baseline (preseizure) values and filtered with a second-order Savitzky–Golay filter with a window length of 7. A window of 40–100 frames was segmented off containing the optical onset of the seizure determined by examining population-wide increases in calcium fluorescence. An onset threshold for each cell was set as the mean + 5*SD of the baseline signal of the 25 frames immediately preceding the beginning of the window. The onset time for each cell was extracted as the frame where the fluorescence amplitude first exceeded the threshold. The recruitment duration was calculated as the time difference between the last detected cellular onset and the first detected cellular onset. The SD of the recruitment durations was calculated over all seizures for all four animals. A total of 17 seizures over four animals were used for the recruitment duration analyses.

For the reliability analyses, we restricted our analyses to seizures captured in common imaging planes; 15 seizures over four animals were used for these analyses (*n* = 3, 4, 2, 6 seizures in each animal). We examined the reliability of the cellular recruitments between seizures by calculating pairwise Spearman’s rank correlation coefficients for the cell rank vectors from pairs of seizures for each animal. We further examined cellular onset reliability by calculating Kendall’s coefficient of concordance between rank vectors obtained from all seizures in each of the four animals. To obtain *p* values for the Kendall’s coefficients, we calculated the Kendall’s coefficients for 1000 randomly shuffled rank matrices, where the cells in each seizure were randomly assigned ranks between 1 and the maximum rank observed for a given seizure. The *p* value for the observed Kendall’s coefficient was determined by calculating the fraction of randomly-generated Kendall’s coefficients that exceeded the observed Kendall’s coefficient.

In three seizures, we calculated vectors to represent the direction of the seizure onset in calcium imaging for later comparison with the direction of the electrophysiological seizure traveling wave (see below). We calculated these vectors as a difference between two points in 2D space, *x2 – x1*. To determine the position of *x1*, we extracted and averaged the locations of all ROIs that had an onset rank of “1” for a particular seizure. To determine the position of *x2*, we extracted and average the locations of all ROIs that had the maximum onset rank for a particular seizure. To transform the vector to anatomic space, we reflected it about the *x-axis* and rotated it 30° toward the *y*-axis to account for rotation of the experimental animal relative to the camera/optical path. We calculated the direction of the seizure onset vectors by calculating the angle of the vector using the MATLAB command “atan2.” A polar histogram of the seizure onset vector directions was plotted with six bins.

#### High γ analyses

Electrophysiological data from annotated seizures was imported into MATLAB, down sampled to 2 kHz, and then bandpass filtered in the 4- to 30-Hz (low frequency) and 80- to 150-Hz (high γ) bands using second-order Butterworth filters. The instantaneous phase of the low-frequency signal and the instantaneous amplitude of the high γ signal were calculated using the Hilbert transform. High γ events were detected on individual channels based on a threshold of a mean + 2.5*SD of the high γ amplitude. The phase of the low-frequency component was extracted at each detected high γ amplitude peak. Polar histograms of the low-frequency phase distributions were plotted with 18 bins. We examined whether the phase distributions of the peak high γ amplitudes differed from randomly selected distributions of phases using the Watson–Williams test in the MATLAB Circular Statistics Toolbox (Berens, 2009). In the case that the assumptions of the Watson–Williams test were violated, we used the multisample test for equal median directions.

#### Multiunit activity (MUA) analyses

Electrophysiological data from annotated seizures was imported into MATLAB and then bandpass filtered in the 4- to 30-Hz (low frequency) and 300- to 3000-Hz (spike) bands using second-order Butterworth filters (Weiss et al., 2013). The instantaneous phase of the low-frequency signal was calculated using the Hilbert transform. MUA was detected by finding negatively-deflecting electrographic events in the spike band that exceeded a threshold, *t*, of:

(1) $$t=-4*median\left(\left|\frac{x\left(t\right)}{0.6745}\right|\right),$$

with *x(t)* as the signal of the spike band (Quiroga et al., 2004). The phase of the low-frequency component was extracted at each detected multiunit event. Polar histograms of the low-frequency phase distributions were plotted with 18 bins. We examined whether the phase distributions of the detected MUA differed from randomly selected distributions of phases using the Watson–Williams test (Berens, 2009). In the case that the assumptions of the Watson–Williams test were violated, we used the multisample test for equal median directions.

#### Traveling wave analyses

To examine the dynamics of the traveling wave, we adapted an analysis first developed to examine traveling waves in seizures across Utah arrays (Liou et al., 2017). In this analysis, we detected the times of individual seizure discharges recorded on the Gr MEAs by detecting peaks in the low-frequency (4–30 Hz) signal that exceeded a threshold of 2 SDs above the mean of the absolute value of the low-frequency signal on individual channels. Peaks detected across channels were considered to be part of the same ictal discharge if they occurred within 150 ms of each other, and only peaks that were detected on at least >60% functional channels were considered for analysis. The peak timing was then associated with the location of the microelectrode on the Gr MEA. Each seizure discharge thus was represented by a set of points (*x*, *y*, *t*) with *x* and *y* as the locations of the channels on the array and *t* as the time of peak amplitude of the low-frequency signal. We then fit a plane for each seizure discharge using these sets of points. We used this fit to calculate the gradient of the plane. We then represented the direction of the wave as the angle of the gradient vector calculated by the inverse tangent, and we calculated the speed of the wave as the inverse norm of the vector. We tested the nonuniformity of the distributions of seizure discharge directions using the Omnibus test (Berens, 2009), and we compared the distributions of seizure discharge directions between animals using the Kolmogorov–Smirnov test (Prism 9). To compare the speeds of the vectors at the beginning and end of the seizure, we extracted the speeds of the vectors in the first 25% of the seizure duration and the vectors in the last 25% of the seizure duration. Twelve seizures were subject to the comparisons of traveling wave speeds in the first 25% of the seizure duration and the last 25% of the seizure duration, for a total of twelve individual comparisons. The number of discharges considered in each test were (no. of discharges in first 25% of duration/no. of discharges in last 25% of duration) 121/121, 101/81, 111/117, 33/34, 129/130, 168/169, 205/206, 46/55, 80/88, 83/90, 109/113, and 125/127.

### Training bootstrap aggregated regression trees for traveling wave speed prediction

#### Feature extraction

For this portion of the study, we restricted our analysis to four seizures from three animals with completely uninterrupted electrophysiology and multicellular calcium imaging data. Many seizures recorded multimodally had brief gaps between imaging time series, as the imaging acquisition was performed by capturing only 3000–6000 frames at a time, whereas electrophysiology was collected continuously at 20 kHz. To avoid the loss of information associated with absent imaging data during transitions between imaging series, only seizures captured entirely within an imaging time series were considered for this analysis. Concatenated multimodal data from these seizures was first imported to MATLAB. This data included (1) calcium fluorescence traces from 10–20 PC *somae* from under the putative site of electrophysiological recording; (2) the electrophysiological recording from the channel above the imaging plane; and (3) the electrophysiological recording from all functional channels in the array. In sliding windows of 4-s duration and 1-s overlap, the following features were calculated from the single channel (above the imaging plane) electrophysiology data: mean high γ amplitude, SD of the high γ amplitude, fractional 4- to 30-Hz power, fractional 80- to 150-Hz power, and the phase locking value (PLV) between the 4- to 30-Hz and 80- to 150-Hz filtered signals. The mean high γ amplitude and the SD of the high γ amplitude were calculated using the amplitude time series derived from the Hilbert transform of the 80- to 150-Hz bandpass filtered (second-order Butterworth) signal in each bin. The fractional band powers were calculated by dividing the integral of the power spectrum over the 4- to 30-Hz or 80- to 150-Hz range by the integral of the entire power spectrum. Finally, PLV was calculated as:

(2) $$PLV=\left|\frac{1}{N}\overset{N}{\underset{n=1}{\sum }}\text{exp}(i\left(\varphi_{4-30Hx}\left[n\right]-\varphi_{a\left(80-150Hz\right)}\left[n\right]\right))\right|,$$

where φ_4–30 Hz_ is the phase of the 4- to 30-Hz bandpass filtered (second-order Butterworth) signal and φ_a(80–150Hz)_ is the phase of the instantaneous amplitude of the 80- to 150-Hz bandpass (second-order Butterworth; Weiss et al., 2013). In sliding windows of 4-s duration and 1-s overlap, the following features were calculated from the multicellular calcium imaging data: mean cellular ΔF/Fo amplitude, mean cellular ΔF/Fo line length, the SD of the first derivative of the cellular ΔF/Fo, mean cell pair ΔF/Fo correlation (Pearson’s *r*), and the SD of the cell pair ΔF/Fo correlations. ΔF/Fo line length was calculated for each cell as:

(3) $$LL=\sum^{}\left|\frac{d}{dt}\Delta F/F_{o}\right|,$$

with d/dt ΔF/Fo as the first derivative of the ΔF/Fo time series (Esteller et al., 2001). In each window, the speed of the traveling wave was set to the median of all traveling wave speed observations in the bin. Bins without traveling wave observations were not considered during model training.

#### Model training and testing

We used features and speeds from three seizures from three different animals as the dataset for model training. We trained 1000 ensembles of 100 bootstrap aggregated regression trees with surrogate decision splits using the curvature test to select the best split predictor. Before model training, we ensured that the training set was equally enriched for features and speeds from all three seizures used in training by randomly selecting bins from the two longest seizures such that the training set consisted of 3*k observations, with *k* observations from each seizure. Models were trained with 5-fold cross validation, and the model that best generalized to a withheld fraction of the training set was stored. The importance of each feature was calculated using the “OOBPermutedPredictorDeltaError” property of the bagged regression tree models in MATLAB. For each variable, the feature importance represents the increase in prediction error if the values of that variable were permuted across the out-of-bag observations. This measure is computed for every tree in the ensemble, averaged over the ensemble, and divided by the SD over the entire ensemble. The performance of the 1000 ensembles was assessed by calculating the Pearson’s correlation coefficient between the experimentally observed speeds from the seizures used in the training set and the predicted speeds from the seizures used in the training set. To examine model generalization, we predicted the traveling wave speed in a fourth, withheld seizure. The generalizability of the 1000 ensembles was assessed by calculating the Pearson’s correlation coefficient between the experimentally observed speeds from the withheld seizure and the predicted speeds from the withheld seizure. The top 100 (10%) performing models were considered the “best generalizing models.” We examined the feature importance, determined as above, in these best generalizing models. For the remaining analyses, we considered the top three most important features in the ensembles of regression trees. The same procedures were performed for 1000 bagged regression trees trained on features derived from only calcium imaging or electrophysiology to compare the performance between single and multimodal models.

#### Most important feature evolution throughout seizure

We then compared the three most important features values between the first 25% versus last 25% of the seizure duration in all seizures used to construct the models. To compare across records, we rescaled the individual features from separate seizures to a range of 0–1, where the minimum feature value was rescaled to 0 and the maximum feature value was rescaled to 1. We then extracted the rescaled feature values from the first 25% of the sliding windows and the last 25% of sliding windows in each seizure and pooled the distributions. We compared the rescaled features from the first 25% of the seizure duration to the last 25% of the seizure duration using Mann–Whitney *U* tests in Prism 9.

## Results

### Multimodal recordings of pharmacologically induced seizures in CA1

**Figure 1.**
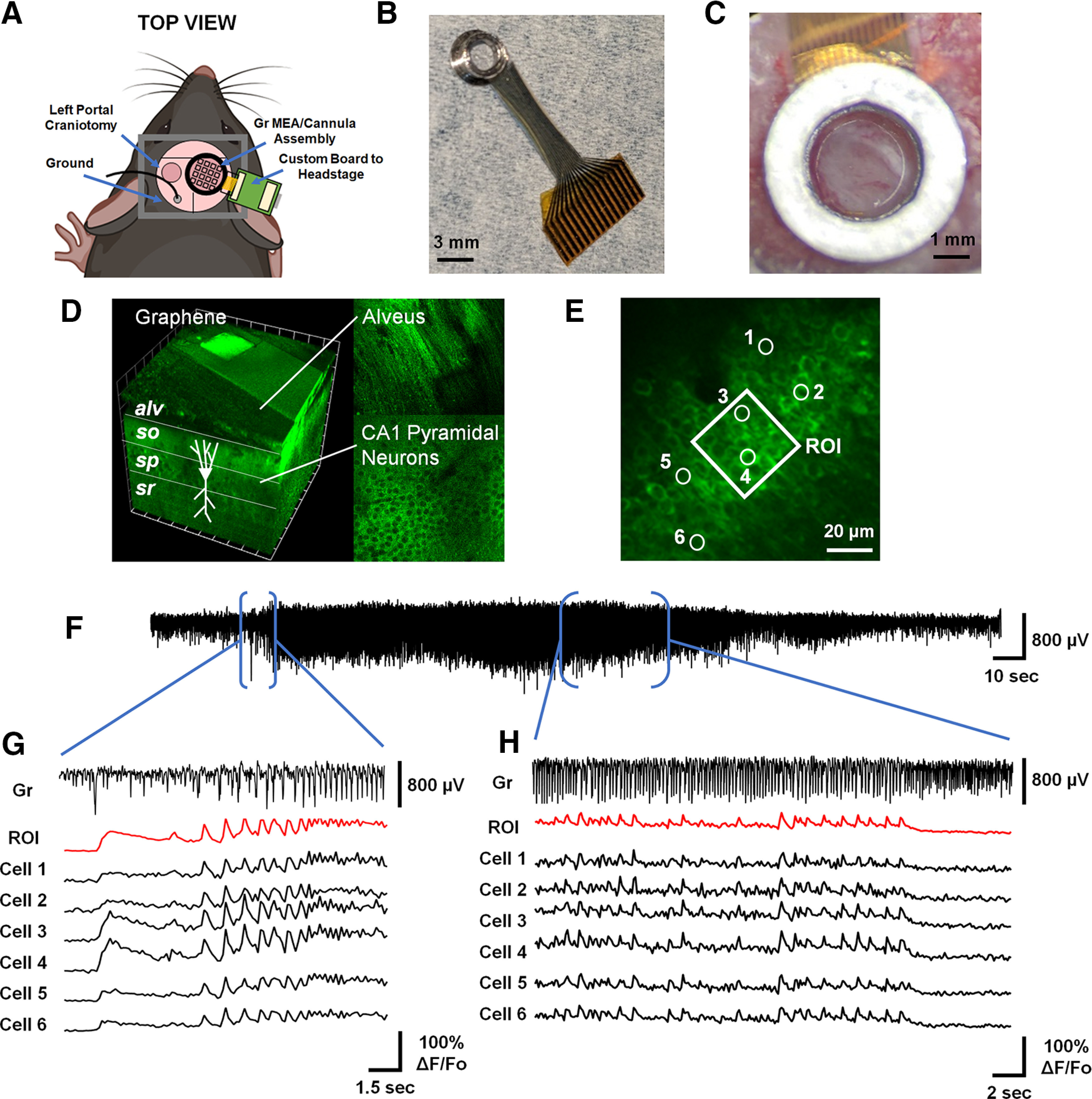
Transparent Gr MEAs enable multimodal recordings of CA1 during acute seizures. ***A***, Overview of the experimental preparation for observing hippocampal seizures. A ground screw is implanted in the left cerebellum, and a bar for head fixation is glued to the skull. A left portal craniotomy is drilled for application of 4-AP to induce seizures. A right craniotomy is drilled and the cortex over the alveus is aspirated to enable implantation of the Gr MEA. Adapted from “Mouse (dorsal)” by BioRender.com. ***B***, The novel implant strategy consisted of a transparent Gr MEA affixed to an imaging window and 3-mm cannula. ***C***, The Gr MEA/imaging window/cannula assembly is implanted over the right alveus fibers. ***D***, This configuration permits two-photon multicellular calcium imaging through the alveus (*alv*) to the layers of dorsal CA1, including stratum oriens (so), stratum pyrimidale (sp), and stratum radiatum (sr) in animals expressing a fluorescent calcium indicator. ***E***, In the sp, two-photon multicellular calcium imaging can resolve individual CA1 PCs. In this schematic, the square ROI corresponds to the approximate area covered by a single 50 × 50 μm Gr microelectrode, and the numbered circles correspond to individual CA1 PCs. ***F***, Following application of 4-AP to the left cortex, seizures later emerge in the right hippocampus (Extended Data Figs. 1-1, 1-2). An example seizure event captured from one Gr microelectrode on the array is displayed. ***G***, The MEA/imaging window/cannula assembly enables simultaneous electrophysiology (labeled Gr) and two-photon multicellular calcium imaging (labeled ROI, cells 1–6, with conventions from panel ***E*** at seizure onset. ***H***, Similarly, simultaneous electrophysiology and two-photon multicellular calcium imaging resolves activity across modalities as seizures evolve.

Understanding seizure activity in the hippocampus *in vivo* has been hampered by limitations to simultaneously leveraging the high temporal fidelity of electrophysiological recording and the high spatial specificity of single-cell neuronal imaging. We designed an experiment to acutely induce seizures and to simultaneously record broadband hippocampal electrophysiology and image individual CA1 PCs *in vivo* in mice expressing a fluorescent calcium indicator (Fig. 1*A*). We developed a novel implant strategy based on a transparent 16-channel Gr MEA affixed to an imaging window and 3-mm cannula (Fig. 1*B*). The MEA consisted of a 4 × 4 grid of 50 × 50 μm square Gr contacts with 500-μm pitch. This assembly was implanted above the alveus over the right dorsal hippocampus of a mouse expressing a genetically encoded fluorescent calcium indicator mainly in PC populations (Fig. 1*C*). Two-photon microscopy through the transparent Gr MEA allowed us to resolve the laminar structure of dorsal CA1 (Fig. 1*D*) and populations of putative CA1 PCs (circular ROIs; Fig. 1*E*) under the site of recording (square ROI; Fig. 1*E*).

10.1523/ENEURO.0386-21.2022.f1-1 Extended Data Figure 1-1 Evolution of right dorsal hippocampal activity following application of 4-AP to the left cortex. In half of our experiments (1/3 Gr MEA implants, 4/7 multishank probe implants), we observed a typical evolution of epileptiform activity in the hippocampus contralateral to the site of 4-AP application to the cortical surface. All traces in Extended Data Figure 1-1 were obtained with multishank probes and from the stratum pyrimidale (sp) of CA1. A, Prior to application of 4-AP to the left cortex, the hippocampus displays low amplitude burst suppression activity. B, 2.5-h trace from sp displaying overview of the evolution of hippocampal activity following 4-AP application to the contralateral cortex. A period of burst suppression evolved to spikes and extended epileptiform patterns in 4/7 animals implanted with multishank probes. Large scale seizures later emerged in 7/7 animals implanted with multishank probes. Emergence of seizures typically was followed by multiple seizures or status epilepticus. The letters above the trace indicate the temporal locations of the activity displayed in panels C–F. C, Burst suppression activity in CA1 persists immediately after application of 4-AP to the left cortex. D, Individual spikes later emerge with a greater amplitude relative to the burst suppression activity. We defined spikes as brief, large amplitude (<1 s, >500 μV) deflections in the envelope of the electrophysiological recording. E, Spikes typically evolved into events characterized by an initial spike with relatively lower amplitude after discharges. These events could endure for up to 30 s and appeared on all recording channels. In this study, we term this type of activity an “extended epileptiform pattern.” We avoid other candidate terminologies, such as interictal polyspikes or microseizure, because the durations of polyspikes or microseizure typically do not exceed 5 s and tend to be confined to restricted areas of the brain. The progression from spikes to extended epileptiform pattern activity was observed in only 4/7 animals implanted with multishank probes. F, Large amplitude, prolonged seizures later emerged in all animals implanted with multishank probes. This activity was the focus of analysis for this study. Download Figure 1-1, TIF file.

**Figure 2.**
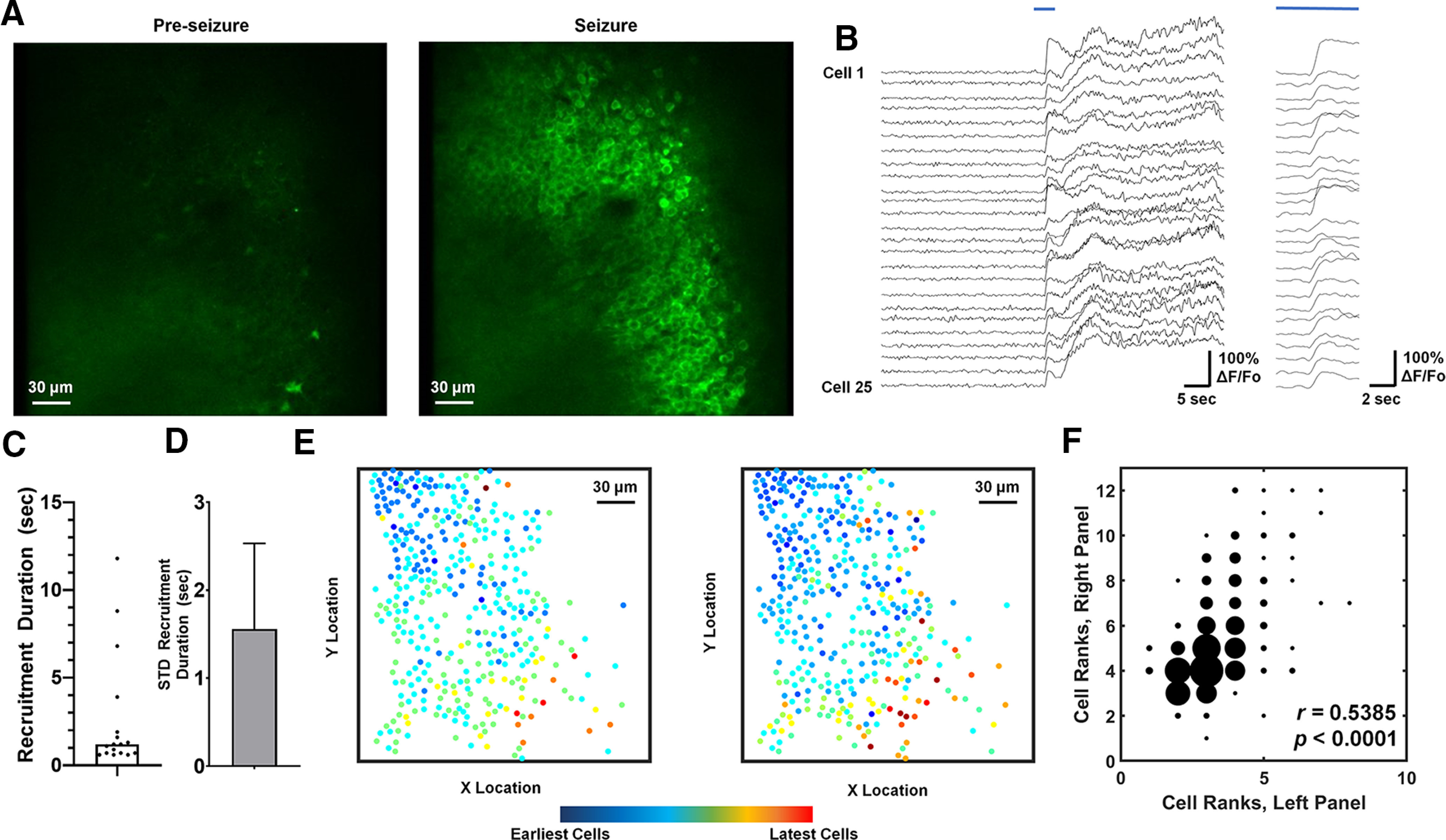
Spatiotemporal characteristics of CA1 PC recruitment to hippocampal seizures. ***A***, Two-photon multicellular calcium imaging during seizure is characterized by a population-wide increase in fluorescence relative to preseizure baseline. ***B***, Representative traces from 25 CA1 PCs at seizure onset (blue line), magnified on the right. ***C***, Distribution of recruitment durations (*n* = 17 seizures, 4 animals). ***D***, Within-animal recruitment duration variability (shown as the mean STD ± SEM). The recruitment duration variability does not change with varying threshold (Extended Data Fig. 2-1). ***E***, Spatiotemporal maps of cell recruitment in two seizures from the same animal, indicating preserved relative recruitments of PCs. Each dot represents the location of a single cell in the imaging frame. The coloration of the dot indicates the relative time of cell recruitment, with blue dots indicating the earliest-recruited cells and red dots indicating the latest-recruiting cells. ***F***, Scatter plot displaying the cell ranks in the seizures displayed in panel ***E***. The marker size varies depending on the number of observations at a coordinate, varying from 1 (smallest marker size) to 27 (largest marker size). The Spearman’s correlation coefficient for this relationship is 0.5385, with *p* < 0.0001.

10.1523/ENEURO.0386-21.2022.f1-2 Extended Data Figure 1-2 Multimodal resolution of epileptiform activity enabled by Gr MEAs. Spike and extended epileptiform pattern activity were observed in 1/3 animals with high quality Gr MEA recordings. A, Representative image obtained using two-photon multicellular calcium imaging in the stratum pyrimidale of CA1. The square ROI corresponds to the approximate area covered by a single 50 × 50 μm Gr microelectrode. B, Representative spike activity, indicated with an asterisk, from a single Gr microelectrode (top) and a corresponding square ROI, indicated in panel A, from the calcium imaging (bottom). C, Representative extended epileptiform pattern activity from a single Gr microelectrode (top) and a corresponding square ROI, indicated in panel A, from the calcium imaging (bottom). Note that the calcium signal reflects a subset of the electrically recorded signal, demonstrating the potential utility of the Gr MEA to uncover how neurons may act together to generate a population signal. Download Figure 1-2, TIF file.

We induced seizures in anesthetized mice by bath applying 4-AP in ACSF (20 μl, 15 mm, 4°C) to the exposed cortical surface contralateral to the site of recording and imaging (Fig. 1*A*). This approach allowed us to capture an hours-long evolution of hippocampal activity as the brain transitioned from a period of ketamine/xylazine-induced burst suppression (Extended Data Fig. 1-1*A–C*) to recurrent seizures. In approximately half of our experiments (1/3 Gr MEA implants, 4/7 multishank probe implants), we observed spikes and extended epileptiform patterns that emerge before seizures (Extended Data Figs. 1-1*D*,*E*, 1–2). All animals exhibited large-amplitude seizures (Fig. 1*F*; Extended Data Fig. 1-1*F*), with the first seizure appearing in the right hippocampus at 1.71 ± 0.49 h (mean ± SD) after application of 4-AP. We restricted our analyses to this activity in this work. Our Gr MEA implant enabled us to concurrently resolve electrophysiological and single cellular calcium fluorescence activity at seizure onset (Fig. 1*G*) and throughout seizure evolution (Fig. 1*H*).

### Dynamics of PC recruitment to recurrent seizures

Relative to baseline, preseizure conditions, seizures were marked by a population-wide increase in calcium fluorescence signal in the field of view (Fig. 2*A*). Extraction of baseline-normalized calcium fluorescence time series from circular ROIs over putative PC cell bodies allowed us to resolve recruitment of individual PCs to seizure activity (Fig. 2*B*).

10.1523/ENEURO.0386-21.2022.f2-1 Extended Data Figure 2-1 Within-animal recruitment duration variability does not change with varying thresholds (ordinary one-way ANOVA, F = 0.006449, p > 0.05, dF = 3.12). Mean STD ± SEM for a threshold set at mean + 2.5, 3, 4, and 5*SD. Download Figure 2-1, TIF file.

Previous multicellular calcium imaging studies in hippocampus *in vitro* and cortex *in vivo* investigated the timescale and reproducibility of cellular recruitment patterns during epileptic activity (Takano et al., 2012; Wenzel et al., 2017). Accordingly, we quantified the absolute recruitment duration and cell recruitment rank reproducibility of optically recorded seizures. We found that the median recruitment duration, defined as the time difference between the first and last detected cellular recruitment time, was 1.2 s (17 seizures captured optically, four animals), with a range of 0.6 s to 11.8 s (Fig. 2*C*). We quantified the within-animal variability in recruitment duration by calculating the SD (STD) of recruitment durations within individual animals. We found that the mean STD of recruitment duration was 1.56 s, with a range of 0–4.29 s (Fig. 2*D*). Importantly, the mean STD of recruitment duration was invariant over a range of thresholds for calcium transient onset (Extended Data Fig. 2-1). We used two measures to examine the reproducibility of PC recruitment to recurrent seizures. The outcome measures were (1) the Spearman’s rank coefficient (*r*) between pairs of seizures within an animal and (2) Kendall’s coefficient of concordance (*W*) among seizures within an animal. Both measures are indicators of ranking order consistency in which values of 0 indicate completely inconsistent recruitment ranks and values of 1 indicate totally consistent recruitment ranks between/among seizures. Of all seizure pairs, 92% of pairs had positive correlations between cell recruitment ranks (23/25 pairs had Spearman’s *r *>* *0 with *p < *0.05; see Materials and Methods). The Spearman *r* between cell recruitment ranks in significantly correlated seizure pairs was 0.489. To visualize an example of the reproducibility of PC recruitment, we display a map of the spatial pattern of relative cell recruitment in two seizures from the same animal in Figure 2*E*. To provide a visualization of the Spearman’s *r* calculation, we display a scatter plot in Figure 2*F* in which the *x*- and *y*-coordinates are the cell ranks between the two seizures displayed in Figure 2*E*. The Spearman’s *r* value for this seizure pair was 0.5385. The consistent recruitment order was also supported by significant Kendall’s *W* values for each animal (one per animal, all significant to *p < *0.05, *W*_1_ = 0.5439, *W*_2_ = 0.3978, *W*_3_ = 0.5671, *W*_4_ = 0.7238).

### Phase-association of high γ and MUA in CA1 during seizure

**Figure 3.**
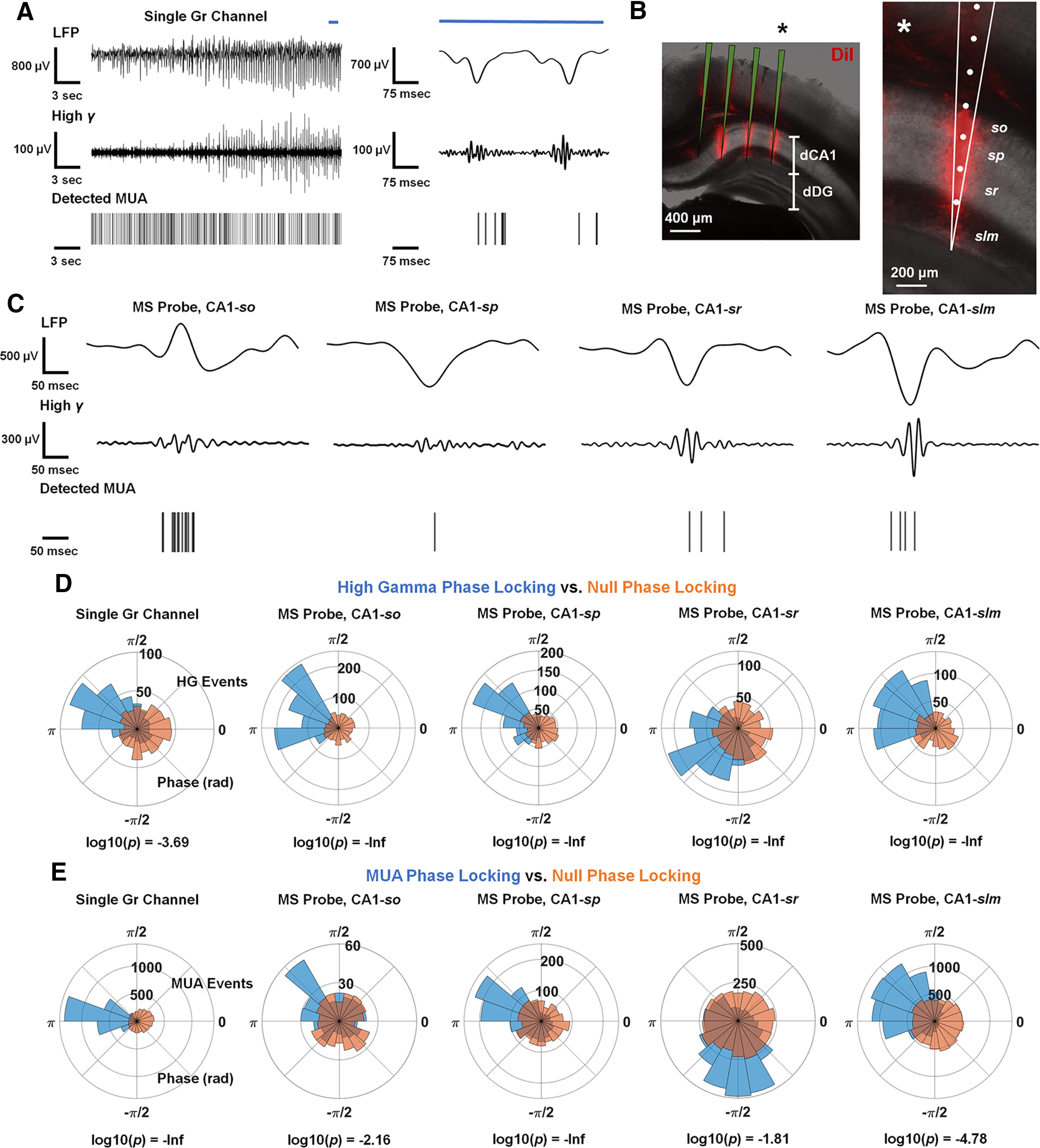
LFP-phase associated high γ and MUA emerge in CA1 during seizure. ***A***, High γ and MUA with phase-association to the low-frequency component of the seizure appear in recordings made with the Gr MEA. Exemplar traces of seizure discharges (shown in the LFP), with concomitant high γ activity and detected MUA. A segment from the established seizure (blue line) is magnified on the right to show the phase-association behavior of γ and MUA (Extended Data Fig. 3-1). ***B***, Location of a multishank probe implanted in dorsal CA1 (dCA1). The dorsal dentate gyrus is labeled as dDG. The placement of the rightmost shank, indicated with an asterisk, is magnified in the right panel. ***C***, High γ and MUA with phase-association to the low-frequency component of the seizure appear in all layers of CA1. Exemplar traces of seizure discharges (shown in the LFP), with concomitant high γ activity and detected MUA. ***D***, Polar histograms of exemplar phase distributions of detected peak high γ amplitudes from a single Gr microelectrode and microelectrodes located in the layers of CA1, shown in blue. Null distributions of randomly selected phase values are shown in orange. Log10-transformed *p* values shown are from the Watson–Williams test or the circular multisample test for equal median directions comparing the two displayed distributions (Extended Data Fig. 3-2). ***E***, Polar histograms of exemplar phase distributions of detected MUA from a single Gr microelectrode and microelectrodes located in the layers of CA1, shown in blue. Null distributions of randomly selected phase values are shown in orange. Log10-transformed *p* values shown are from the Watson–Williams test or the circular multisample test for equal median directions comparing the two displayed distributions.

Beyond seizure onset, a hallmark feature of brain territories recruited to seizure is the presence of high γ and MUA phase-locked to the local field potential (LFP; Schevon et al., 2012; Weiss et al., 2013, 2015). While the saturation and low sampling frequency of the calcium imaging data limited our ability to resolve activity beyond seizure onset, the high temporal resolution of electrophysiology allowed us to examine and analyze fast activity during seizures. When we filtered the data obtained from the Gr MEA in the LFP (4–30 Hz), high γ (80–150 Hz), and spike (300–3000 Hz) bands, we noted the presence of high γ and MUA with strong phase association to the LFP. To confirm that the signals recorded using the Gr MEA on the hippocampal surface reflected activity within the CA1 network, we examined data recorded with multishank probes targeted to dorsal CA1, which enabled layer-specific resolution of high γ and MUA (Fig. 3*B*; Extended Data Fig. 3-1). We found that high γ and MUA displayed phase-association to the LFP in all layers of CA1 (Fig. 3*C*). To corroborate these observations, we found that the observed distributions of LFP phase values associated with detected high γ activity peak amplitudes were significantly different from randomly selected distributions of LFP phase values of equal size in both Gr MEA and multishank probe recordings (Fig. 3*D*). Similarly, we found that the observed distributions of LFP phase values associated with detected MUA were significantly different from randomly selected distributions of LFP phase values of equal size in both Gr MEA and multishank probe recordings (Fig. 3*E*). These observations were recapitulated in substantial fractions of Gr MEA and multishank probe channels over all seizures recorded (Extended Data Fig. 3-2).

10.1523/ENEURO.0386-21.2022.f3-1 Extended Data Figure 3-1 Representative MUA recorded during a single seizure discharge using a Gr MEA. The traces are derived from the LFP (4–30 Hz) and the spike band (300–3000 Hz). In the bottom panel, single lines indicate time points with detected MUA. Download Figure 3-1, TIF file.

10.1523/ENEURO.0386-21.2022.f3-2 Extended Data Figure 3-2 Histograms of p values comparing observed high γ peak amplitude and multiunit phase distributions relative to null distributions. Note that all histograms have log10-transformed y-axes. This choice was made because all p values that were equal to 0 were manually reset to 0.01 for plotting purposes, and the samples became highly enriched at p = 0.01. All bars to the left of the red line, indicating p = 0.05, were considered significant. P values were obtained from the Watson–Williams test or the circular multisample test for equal median directions comparing the experimentally observed phase distribution and a randomly sampled phase distribution. A, Histogram of p values for comparisons of phase distributions of peak high γ amplitude versus null distributions from the Gr MEA. 100% (106/106) of channels over all seizures recorded displayed phase distributions of high γ peak amplitude that differed from null distributions. B, Histogram of p values for comparisons of phase distributions of peak high γ amplitude versus null distributions from the multishank probes. 89.2% (215/241) of channels over all seizures recorded displayed phase distributions of high γ peak amplitude that differed from null distributions. C, Histogram of p values for comparisons of phase distributions of MUA versus null distributions from the Gr MEA. 92.5% (98/106) of channels over all seizures recorded displayed phase distributions of MUA that differed from null distributions. D, Histogram of p values for comparisons of phase distributions of MUA versus null distributions from the multishank probes. 92.1% (222/241) of channels over all seizures recorded displayed phase distributions of MUA that differed from null distributions. Download Figure 3-2, TIF file.

### Seizure discharges form traveling waves through the CA1 network

**Figure 4.**
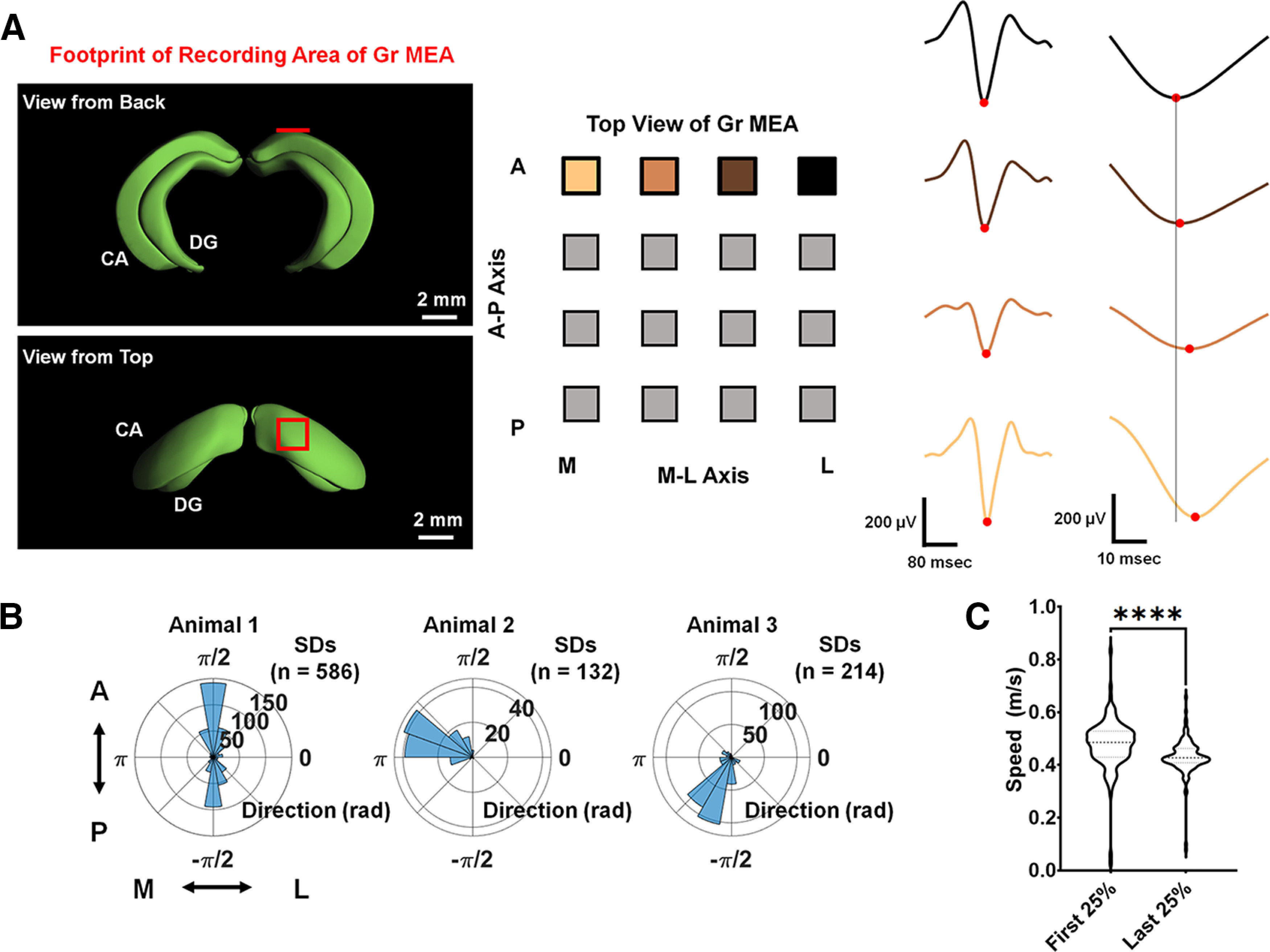
The low-frequency component of the seizure discharge is a traveling wave with variable direction and speed. ***A***, The peak amplitude of the seizure discharge has variable latency across the Gr MEA. Left, Anatomical location of the recording area of the Gr MEA relative to the cornu ammonis (CA) and dentate gyrus (DG). Anatomical images courtesy of the Allen Brain Atlas 3D Viewer. Center, The footprint of the Gr MEA corresponding to the seizure discharge shown at right, with four electrode positions color coded as in the LFP trace. The horizontal axis corresponds to the medial-lateral (M-L) axis, with the most lateral channels located on the right of the panel, and the vertical axes corresponds to the anterior-posterior (A-P) axis, with the most anterior channels located on the top of the panel. Right, Recording of one representative discharge from four channels on a Gr MEA, with the peak amplitudes indicated with red dots. The earliest discharge is in black, and later discharges are in lighter colors. The rightmost portion of the panel displays a magnified view of the peak, with a vertical line to guide the eye and highlight the differences in peak latencies. ***B***, Distributions of traveling wave directions in the three animals with high-quality Gr MEA recordings, displayed as polar histograms. In these plots, the angle corresponds to the direction of the gradient vector, and the radius corresponds to the number of seizure discharges (SDs) observed in the seizure. The arrow guide adjacent to the left most polar histogram provides an orientation of the plots along the A-P and M-L axes. (Extended Data Figs. 4-1, 4-2). ***C***, Violin plots displaying the distributions of discharge speeds in the first 25% of a single seizure relative to the last 25% of a single seizure. The speed of the traveling wave decreases significantly in the last 25% of the seizure relative to the first 25% of the seizure (Mann–Whitney *U* test, *U *= 4799, ****p <* *0.0001 for the comparison shown).

Beyond the scales of single and ensembles of cells, network dynamics are thought to be important drivers of seizure activity (Schevon et al., 2012; Martinet et al., 2017; Driscoll et al., 2021). In addition to enabling imaging of local populations of CA1 PCs and recording at single sites on the surface of CA1, the Gr MEA configuration allowed us to resolve network activity during seizures. We examined the propagation of individual seizure discharges across the CA1 surface in the Gr MEA recordings. Analysis of individual seizure discharges in the LFP robustly revealed variable latencies across the Gr MEA (Fig. 4*A*). To quantify the speed and direction of the traveling wave, we modified an analysis originally developed to map traveling waves across Utah array recordings from human epilepsy patients. In this paradigm, each seizure discharge was treated as a 3D data point (*x*, *y*, *t*), where the *x*- and *y*-coordinates corresponded to the spatial location in the 2D Gr MEA plane and t corresponded the time of the peak LFP amplitude during a seizure discharge. We grouped data points based on their temporal proximity to each other (see Materials and Methods) to capture the propagation of single discharges across the MEA. The traveling wave direction was determined by the gradient direction of the plane, and the traveling wave speed was determined by the inverse of the gradient norm.

10.1523/ENEURO.0386-21.2022.f4-1 Extended Data Figure 4-1 Traveling wave directions of all seizures captured electrographically on the Gr MEA. All panels are oriented in the same convention as Figure 4C. As a reminder, the orientation of the angles along the A-P and M-L axes are displayed in the leftmost polar histogram of panel A. A, Distributions of the traveling wave direction for other seizures (i.e., those seizures not pictured in Fig. 4C) captures using the MEA from animal 1. B, Distributions of the traveling wave direction for other seizures captures using the MEA from animal 2. C, Distributions of the traveling wave direction for the other seizures captures using the MEA from animal 3. Download Figure 4-1, TIF file.

10.1523/ENEURO.0386-21.2022.f4-2 Extended Data Figure 4-2 Consistency of directions of electrophysiological seizure traveling waves and three seizure onsets captured via calcium imaging. Both panels are oriented in the same convention as Figure 4C and Extended Data Figure 4-1. A, Sample distribution of the traveling directions for a seizure captured using the MEA from animal 1. B, Polar histogram showing the distribution of the directions of seizure onsets (SOs) captured using multicellular calcium imaging. Comparisons of traveling wave speeds between modalities are not included due to the relatively slow calcium imaging sampling frequencies of 5 Hz used in this study. Download Figure 4-2, TIF file.

In the three animals with high quality Gr MEA recordings, we found that the direction of the seizure traveling wave was highly non-uniform (*p* < 0.0001 by the Omnibus test; Fig. 4*B*) and was distributed differently among animals (*p* < 0.0001 by pairwise Kolmogorov–Smirnov tests; Fig. 4*B*). While there was significant inter-animal variability, the traveling wave directions between seizures were highly consistent within individual animals (Extended Data Fig. 4-1). Moreover, we found that the directions of seizure onset captured via calcium were consistent with the distributions of seizure traveling wave direction (Extended Data Fig. 4-2). To examine the evolution of the traveling wave speed throughout the seizure, we compared the distributions of seizure discharge speeds from the first 25% of discharges versus the last 25% of discharges using a Mann–Whitney *U* test for each individual seizure (Fig. 4*C*). In the majority (9/12) of individual seizures, we found that the last 25% of discharges had a significantly lower median speed compared with the first 25% of discharges in a seizure. In the figure shown, the median speed in the first 25% of discharges was 0.4856 m/s, and the median speed in the last 25% of discharges was 0.4273 m/s. In the remaining 3/12 individual seizures, no difference was uncovered between the two distributions of speeds.

### Relating single site, multimodal features to the traveling wave speed via bootstrap aggregated regression trees

**Figure 5.**
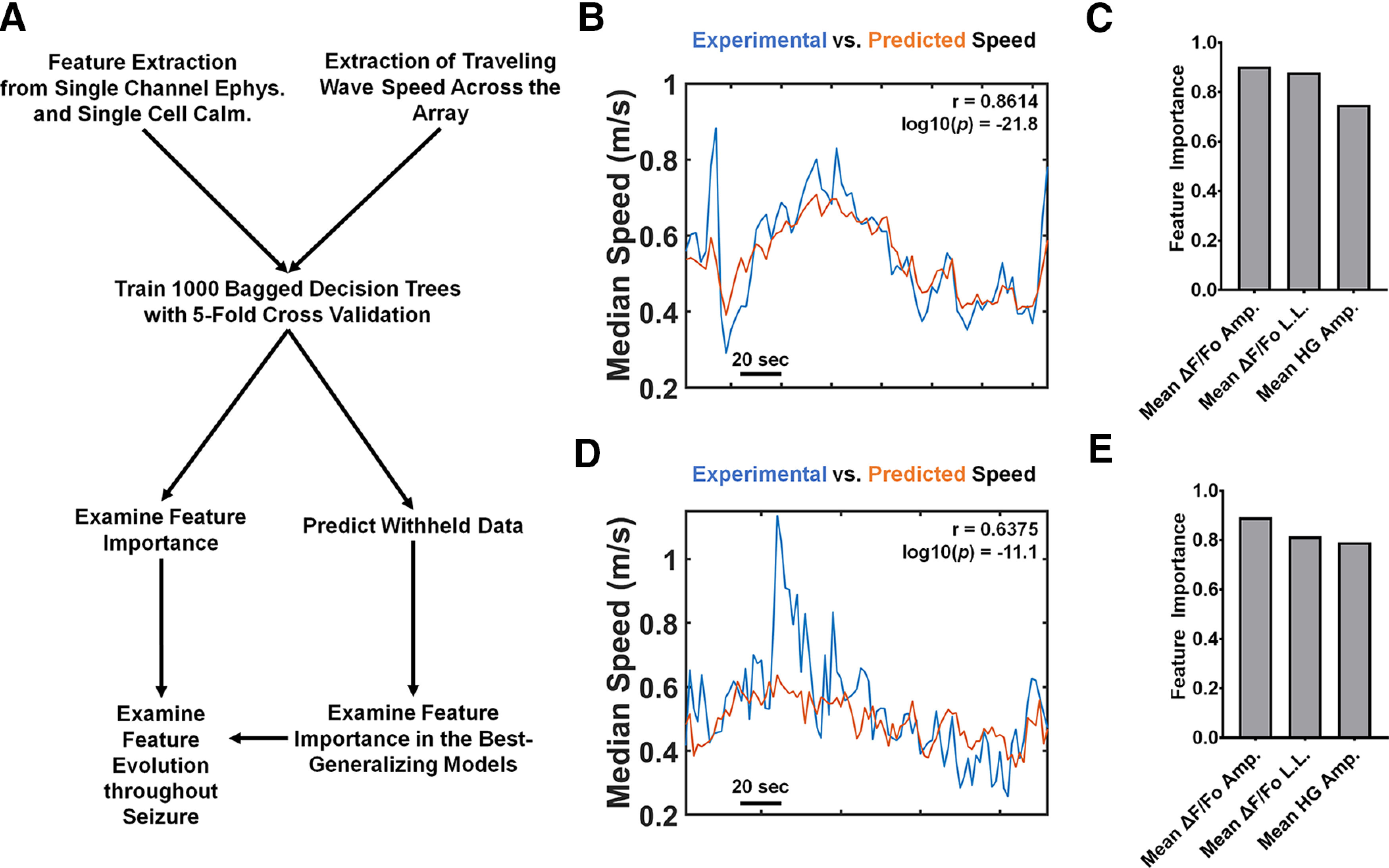
Prediction of seizure discharge traveling wave speed with single site, multimodal features using bootstrapped aggregated (bagged) regression trees. ***A***, Simplified workflow detailing the machine learning experiment. Following the extraction of multimodal features from single channel electrophysiology and multicellular calcium imaging and the extraction of the traveling wave speed across the Gr MEA, we trained 1000 bagged regression trees with 5-fold cross validation. We examined feature importance across the 1000 models, and after predicting the traveling wave speed in a withheld dataset, examined feature importance in the best-generalizing models (Extended Data Fig. 5-1). Regression trees trained on multimodal features outperformed trees trained on single modality features (Extended Data Fig. 5-2). ***B***, Prediction of traveling wave speed in a single seizure included in the training dataset. The experimentally observed traveling wave speed is displayed in blue, and the predicted traveling wave speed is displayed in orange. The Pearson’s *r* value between the two series is 0.8614, with log10(*p*) = −21.8. ***C***, Bar plot displaying the three most important features in the population of 1000 bagged regression trees. A representative image of the time evolution of the three most important features throughout a seizure is displayed in Extended Data Figure 5-3. In descending order of importance, they are the mean cellular ΔF/Fo amplitude (mean ΔF/Fo amp.), mean cellular ΔF/Fo line length (mean ΔF/Fo L.L.), and the mean high γ amplitude (mean HG amp.; Extended Data Fig. 5-1). ***D***, Prediction of traveling wave speed in a seizure withheld from the training dataset. The experimentally observed traveling wave speed is displayed in blue, and the predicted traveling wave speed is displayed in orange. The Pearson’s *r* value between the two series is 0.6375, with log10(*p*) = −11.1. ***E***, Bar plot displaying the three most important features in the 100 best (top 10%) generalizing bagged regression trees. In descending order of importance, they are the mean cellular ΔF/Fo amplitude (mean ΔF/Fo amp.), mean cellular ΔF/Fo line length (mean ΔF/Fo L.L.), and the mean high γ amplitude (mean HG amp.; Extended Data Fig. 5-1).

Our technique allows us to observe hippocampal activity across two modalities (calcium imaging and electrophysiology) and multiple scales (single cells, cell ensembles, and network activity). To synthesize these data, we examined whether we could predict the traveling wave speed across individual seizures using features derived from single site electrophysiology and imaging of individual PCs under the putative site of recording. Towards this end, we extracted 10 time-varying features (five features per modality, 4-s duration sliding window with 1-s overlap) from four seizures with uninterrupted multimodal data (Materials and Methods). We trained 1000 bootstrap aggregated (bagged) regression tree ensembles on data from three of the four seizures to predict traveling wave speed from single site multimodal features (Fig. 5*A*). We found that the 1000 bagged regression trees could predict the traveling wave speed in the training datasets to a median Pearson’s *r* of 0.7699 between experimental and predicted median speed time series (Fig. 5*B*). The most important features in the 1000 bagged regression tree ensembles were mean fluorescence amplitude, mean calcium imaging line length (a measure of signal complexity), and mean high γ amplitude (Fig. 5*C*; Extended Data Fig. 5-1). When we made predictions on a fourth, withheld, seizure, we found that the top 10% of generalizable bagged regression trees predicted the traveling wave to a median Pearson’s *r* of 0.6208 between experimental and predicted median speed time series (Fig. 5*D*). The most important features in the best-generalizing models were mean fluorescence amplitude, mean calcium imaging line length, and mean high γ amplitude (Fig. 5*E*; Extended Data Fig. 5-1). Furthermore, we found that the trees trained on multimodal features outperformed trees trained on features derived from only one modality in predicting training and withheld data (Extended Data Fig. 5-2). The median Pearson’s *r* values between predicted and true training traveling wave data were 0.7322 and 0.7127 for trees trained on calcium imaging and electrophysiology only features, respectively. These values differed significantly compared with the median Pearson’s *r* of 0.7699 for predicting training and withheld data in multimodal regression trees (Extended Data Fig. 5-2*A*). For the top 100 generalizing models, the median Pearson’s *r* values between true and predicted withheld traveling wave data were 0.4824, 0.5880, and 0.6208 for calcium imaging only, electrophysiology only, and multimodal regression trees (Extended Data Fig. 5-2*B*). The performance of the different models in predicting withheld data all different significantly to *p *<* *0.01.

10.1523/ENEURO.0386-21.2022.f5-1 Extended Data Figure 5-1 Feature importance values in 1000 bagged regression trees and the top 10% generalizing models. The three most important features are indicated in bold. Download Figure 5-1, DOC file.

10.1523/ENEURO.0386-21.2022.f5-2 Extended Data Figure 5-2 Regression trees trained on multimodal features outperform trees trained on features derived from single-modal features. ***A***, Performance of models trained on single modal and multimodal features to predict traveling wave speeds in the training dataset. The performance was measured as the Pearson’s *r* between the true and predicted traveling wave values. Here, the mean Pearson’s *r* over 1000 model predictions for the three different model types is shown, and the error bar represents the SD. The three groups all have medians that differ from each other (Kruskal–Wallis test, K-W statistic = 1640, *p *<* *0.0001, Dunn’s multiple comparisons tests, *p *<* *0.0001 for all). ***B***, Performance of top 100 best-performing models trained on single modal and multimodal features to predict traveling wave speeds in a dataset withheld from model training. Here, the mean Pearson’s *r* over 100 model predictions for the three different model types is shown, and the error bar represents the SD. The three groups all have medians that differ from each other (Kruskal–Wallis test, K-W statistic = 180.4, p < 0.0001, Dunn’s multiple comparisons tests, p < 0.01 for all). Download Figure 5-2, TIF file.

10.1523/ENEURO.0386-21.2022.f5-3 Extended Data Figure 5-3 Representative time evolution of rescaled values of the three most important features for one seizure. The entire seizure duration shown is 330 s. Download Figure 5-3, TIF file.

**Figure 6.**
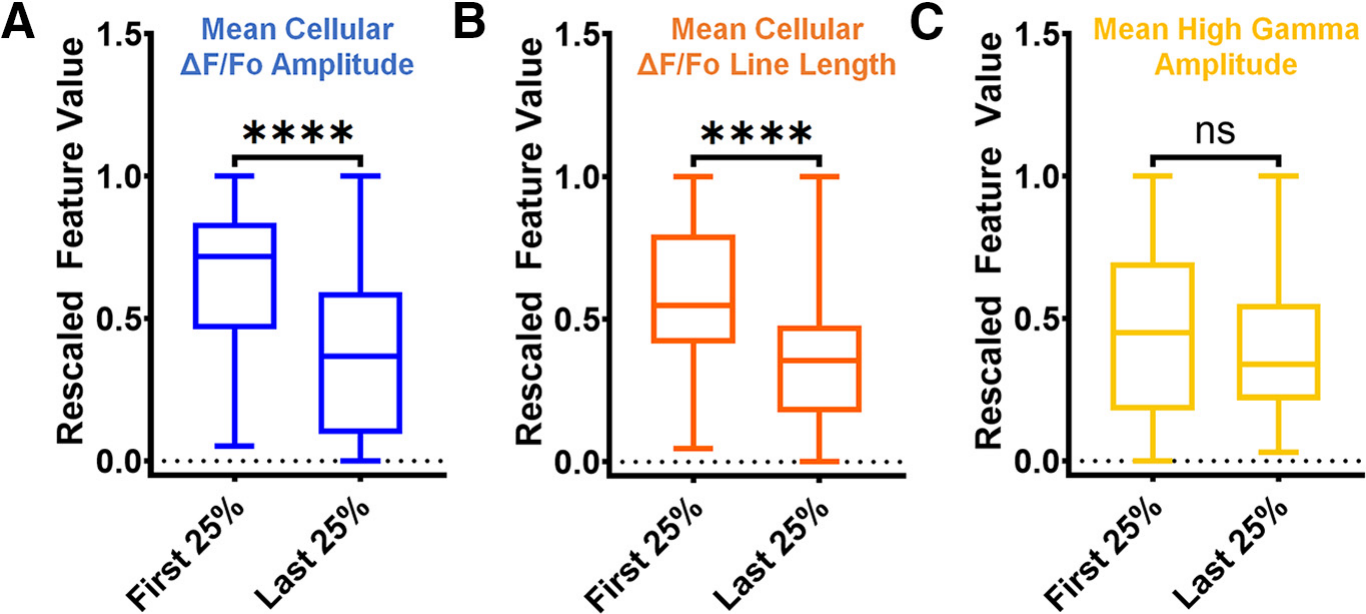
Comparison of pooled (*n* = 3 seizures), rescaled values of the three most important features in the first 25% of seizure duration versus the last 25% of seizure duration. ***A***, Mean cellular ΔF/Fo amplitude significantly decreases in the last 25% of seizure duration relative to the first 25% (Mann–Whitney *U* test, *U *=* *1886, ****p <* *0.0001). ***B***, Mean cellular ΔF/Fo line length significantly decreases in the last 25% of seizure duration relative to the first 25% (Mann–Whitney *U* test, *U *=* *2159, ****p <* *0.0001). ***C***, Mean high γ amplitude does not differ in the last 25% of seizure duration relative to the first 25% (Mann–Whitney *U* test, *U *=* *3545, ns*p *>* *0.05). A summary of all analyses can be found in Extended Data Figure 6-1.

Recalling that the speed of the traveling wave tends to be slower in the last 25% of discharges relative to the first 25% (Fig. 4*C*), we hypothesized that the three most important features would differ in the first 25% versus the last 25% of speed bins. We rescaled the mean fluorescence amplitude, mean calcium imaging line length, and mean high γ amplitude in these three seizures (Extended Data Fig. 5-3) and pooled the features from the first 25% versus the last 25% of each seizure. We found that the mean fluorescence amplitude was substantially lower in the last 25% of the seizure relative to the first 25% (*p *<* *0.0001 by the Mann–Whitney *U* test; Fig. 6*A*). Similarly, we found that the mean calcium imaging line length was substantially lower in the last 25% of the seizure relative to the first 25% (*p *<* *0.0001 by the Mann–Whitney *U* test; Fig. 6*B*). Finally, we uncovered no difference between the mean high γ amplitude in the two epochs (Fig. 6*C*).

10.1523/ENEURO.0386-21.2022.f6-1 Extended Data Figure 6-1 Summary of analyses presented in this work relative to seizure evolution. Download Figure 6-1, TIF file.

## Discussion

### Experimental considerations

Performing simultaneous, multimodal measurements of neural activity remains a substantial challenge in neuroscience. Combining modalities can provide different perspectives on, and thus a more comprehensive view of, neural activity (Lu et al., 2021). Moreover, multimodal measurements can overcome the drawbacks of single modalities and enable observations across scales, from single cells to networks. A summary of the analyses of our study can be found in Extended Data Figure 6-1. Our study was made possible by optically transparent MEAs based on graphene. First developed in 2014, multimodal measurements with Gr MEAs have been demonstrated in hippocampal slices *in vitro* (Kuzum et al., 2014) and *in vivo* in mouse cortex with low-magnification calcium imaging (Driscoll et al., 2021). We adapt the same MEA technology used in Driscoll’s study by incorporating it into a cannula/imaging window assembly, and we implant it in the hippocampus, rather than cortex. Our study represents an advance relative to this previous work as we performed two-photon multicellular calcium imaging of defined neuronal populations in CA1 and extracellular electrophysiology of neural ensembles and the CA1 network. Critically, this multimodal paradigm enables us to monitor seizure activity across these scales.

Consistent with previous *in vivo* seizure imaging studies in cortex, we induced seizures in anesthetized animals during our experiments (Wenzel et al., 2017, 2019; Liou et al., 2018). Performing experiments under anesthesia reduced the animal burden of an hours-long evolution of epileptic activity. Indeed, anesthesia may change an animal’s seizure threshold or seizure propagation speed relative to wakefulness, but importantly, seizures in humans also may occur under deep anesthesia in the operating room or the medical intensive care unit (Wenzel et al., 2017, 2019). To induce seizures in our experiments, we bath applied 4-AP to the cortical surface contralateral to the site of hippocampal recording and imaging. Inducing seizures in this manner minimized disturbances to the objective lens of the two-photon microscope. After applying 4-AP contralateral to the site of imaging and recording, we observed epileptiform activity including spikes and extended epileptiform patterns in half of our experiments, consistent with clinical observations that epileptiform activity in patients with MTLE may occur hours before the clinical onset of seizures (Litt et al., 2001). Furthermore, when the hippocampus seized, we observed seizures of hypersynchronous onset, a typical pattern of seizure onset in MTLE patients (Weiss et al., 2016). One important caveat of our model is that while CA1 may receive seizure-promoting input from the entorhinal cortex or CA3, the global site of seizure induction is not in the hippocampal formation or the extended limbic system. The global site of seizure induction is the cortex contralateral to the hippocampus under recording and imaging. Models exploiting focal uncaging/application of convulsants (Miri et al., 2018) or stereotaxically targeted electrical kindling (Takeuchi et al., 2021) may better recapitulate this feature of MTLE, although seizure initiation sites within single animals are highly variable in naturalistic models of MTLE (Toyoda et al., 2013).

### Hippocampal versus cortical seizures

While hippocampal seizures *in vivo* have been understudied, our understanding of focal cortical seizures has advanced rapidly in the last 15 years (Schevon et al., 2019). Our study reports multiple observations convergent with observations made in animal models and human recordings of focal cortical seizures. As has been observed in PCs of the cortex, we report that PCs in CA1 exhibit variable, or elastic, absolute recruitment durations and reliable patterns of recruitment between seizures in individual animals (Wenzel et al., 2017, 2019). The relatively slow recruitment (1.2 s on average to traverse a ∼300-μm field, with speeds on the order of hundreds of μm/s) observed at seizure onset in multicellular imaging resembled the evolution of a putative ictal core territory previously shown in low-magnification calcium imaging experiments (Schevon et al., 2012, 2019; Liou et al., 2020; Driscoll et al., 2021). In addition, we find that high γ and MUA organizes around the phase of the low-frequency seizure discharge in all layers of CA1 (Schevon et al., 2012; Weiss et al., 2013, 2015). Measures derived from these band-specific activities, such as the “phase-locked high γ” measure, have been proposed as biomarkers to determine cortical regions for resection during epilepsy surgery (Weiss et al., 2015).

At the network level, we observe highly non-uniform distributions of seizure discharge traveling wave directions, as has been reported in human cortical epilepsy recordings (Smith et al., 2016; Martinet et al., 2017). The speed of the seizure discharge traveling wave (on the order of tens of cm/s) was orders of magnitude faster than the speed of seizure onset propagation observed in calcium imaging, which is consistent with experimental data from human epilepsy recordings by Schevon et al. (2012) and the computational model of seizure propagation proposed by Liou et al. (2020). In our experiments, the distributions of the traveling wave direction differed between animals. This observation may reflect animal-specific differences in network connectivity. Indeed, previous work has suggested that neurons in the epileptic hippocampus that are highly correlated during non-seizure periods may form a strongly connected network that facilitates the propagation of epileptic activity (Neumann et al., 2017). One important divergent observation between our study and the cortical seizure literature is that the traveling wave tends to decrease in speed in the final 25% of the seizure duration relative to the first 25% of the seizure duration. At least two studies analyzing electrophysiological data from human cortical epilepsy patients reported an increase in the speed of the traveling wave as the seizure approaches termination (Smith et al., 2016; Liou et al., 2017). In cortical seizures, the increase in the traveling wave speed late in seizure is thought to reflect the instability of an “ictal wavefront.” This wavefront is a sharply demarcated (<2 mm wide) region of the cortex with continuous multiunit firing that gives rise to rhythmic discharges across broad areas of the cortex and whose input sharply dissipates at seizure termination (Schevon et al., 2019). This difference between observed cortical and hippocampal traveling wave speeds might be because of regional differences in cellular and network architecture, species differences between rodent and human brains, or our pharmacological model of epilepsy. Nonetheless, our observation may be the result of a distinct network process that occurs at seizure termination in CA1 of the hippocampus.

### Relating single-site multimodal features and array-wide traveling wave speed

While our implant strategy enabled observations of seizures at the levels of single PCs, ensembles of cells, and the CA1 network, relating observations of neural activity across scales remains a substantial conceptual problem (Lu et al., 2021). Observing neural activity across scales allows for a more complete picture of the neural processes behind phenomena like seizures. Indeed, the advance of multimodal recording technologies requires a concomitant advance in the analytical techniques used to understand multimodal, multiscale data (Driscoll et al., 2021). We used a machine learning-based approach to synthesize our analyses of the multimodal data across multiple scales of neural activity. We used bagged regression trees to predict the speed of the seizure traveling wave from features derived from single channel electrophysiology and multicellular calcium imaging. We chose regression trees as our model paradigm because of the well-established nonlinear nature of multiscale phenomena in the brain (Eissa et al., 2017). Using bagged regression trees improved the likelihood of the models generalizing to data from a fourth seizure withheld from the training dataset. Yet, bagged regression trees remained interpretable by way of examining the feature importance across all models trained (Fig. 5*C*) and the best generalizing models (Fig. 5*E*). Importantly, bagged regression trees trained on multimodal features outperformed trees trained on features from only one modality (either calcium imaging or electrophysiology) in the prediction of both training and withheld data (Extended Data Fig. 5-2). This result provides direct evidence for the importance of combining features from multimodal modalities to construct interpretable models.

We hypothesized that examining the values of the most important features in the first 25% versus last 25% of time bins might reveal differences reflective of physiological changes related to the slowing of the traveling wave speed. Importantly, this hypothesis does not imply that a change in feature values has a direct causal relationship with the slowing of the seizure discharge traveling wave speed *in vivo*. The most important features were mean cellular ΔF/Fo amplitude, mean cellular ΔF/Fo line length, and mean high γ amplitude. Mean cellular ΔF/Fo amplitude reflects how much the cells fluoresce in a given window. Although the intracellular calcium levels during a seizure likely exceed the linear regime relating action potentials and calcium transients, changes in ΔF/Fo amplitude are likely reflective of changes in somatic calcium levels in PCs. Cellular ΔF/Fo line length is a feature calculated as the sum of the absolute value of the first derivative of the ΔF/Fo time series. The first derivative of the ΔF/Fo time series is sensitive to changes in the ΔF/Fo time series and is commonly used to detect calcium transient events (Trevelyan et al., 2006). This feature is sensitive to fluctuations in signal amplitude and signal frequency (Esteller et al., 2001). Finally, mean high γ amplitude is directly reflective of the amplitude of the 80- to 150-Hz filtered electrophysiological signal at a given time point.

Between the traveling wave speed analysis (Fig. 4*D*) and the feature analyses (Fig. 6), we noted four co-incident observations between the first 25% and last 25% of the seizure duration: the traveling wave speed tends to decrease, the mean cellular ΔF/Fo amplitude feature values decrease, the mean cellular ΔF/Fo line length feature values decrease, and the high γ amplitude values remain the same. To interpret this result, we noted multiple convergent threads of evidence from the seizure literature and our experiments. (1) Discharge traveling wave speed in models of cortical seizures is controlled by feedforward inhibition: epileptiform activity propagates slowly when inhibition is intact and quickly when inhibition is compromised (Trevelyan et al., 2006, 2007). (2) Synaptic inhibition from parvalbumin (PV–) and somatostatin-expressing (SST–) interneurons remains intact through the preseizure and early seizure phases (Miri et al., 2018). (3) Fast-spiking interneurons are thought to fire in structured temporal sequences during individual seizure discharges in the epileptic hippocampus (Neumann et al., 2017). (4) In our multishank probe experiments, we noted the presence of phase-associated multiunit firing and high γ activity in the interneuron-dominant strata oriens, radiatum, and lacunosum moleculare of CA1 (Fig. 2*C*). Taken together, the co-incidence of the reduction of traveling wave speed, reduction in calcium imaging feature values, and maintenance of the high γ amplitude may represent a late-seizure, local inhibition-dominant network state that restrains the seizure and facilitates to the transition to the postseizure state.

### Limitations and outlook

Our results suggest a dynamic interplay between PC activity and the inhibitory network of CA1 in the transition from seizure to a postseizure state. A natural extension of this work is thus to induce seizures and perform simultaneous electrophysiological recordings and multicellular calcium imaging experiments in mice with calcium indicators targeted to genetically defined populations of hippocampal interneurons. Furthermore, using chronic seizure models that recapitulate the underlying pathophysiology and state dependence of seizure occurrence in MTLE may offer more naturalistic views of seizure dynamics (Ewell et al., 2015; Kahn et al., 2019).

Ultimately, three important themes emerge from the analyses of our study. First, our Gr MEA/imaging window/cannula assembly enables otherwise intractable cross-modal, cross-scale measurements in CA1 of the hippocampus. Next, hippocampal and cortical seizures may exhibit similar phenomena at the scales of individual cells and neural ensembles but different phenomena at the network scale. Finally, drawing on the results of our data reduction and machine learning experiments, CA1 may transition among states as seizures evolve toward termination.

## Synthesis

Reviewing Editor: William Stacey, University of Michigan Decisions are customarily a result of the Reviewing Editor and the peer reviewers coming together and discussing their recommendations until a consensus is reached. When revisions are invited, a fact-based synthesis statement explaining their decision and outlining what is needed to prepare a revision will be listed below. The following reviewer(s) agreed to reveal their identity: Tristan Shuman, Kyle Lillis. Note: If this manuscript was transferred from JNeurosci and a decision was made to accept the manuscript without peer review, a brief statement to this effect will instead be what is listed below.

In this manuscript, the authors performed 2-photon calcium imaging and graphene MEA-based electrophysiological measurements, sometimes synchronously during chemically induced seizures. The primary findings were:

1. Neuronal recruitment into seizure discharges is consistent at the cellular level

2. Surface and depth electrodes show strong phase association between MUA and high gamma vs LFP.

3. Seizures form travelling waves with a highly repeatable direction

4. The speed of wave propagation decreases during the seizure, which can be predicted from single-site multimodal (calcium + ephys) data.

Overall, this manuscript describes a technically interesting recording configuration wherein high spatial resolution calcium imaging recordings are combined with high temporal resolution electrophysiological recording (using transparent graphene electrode arrays). The findings relating single site multimodal data to large scale ictal wave propagation are interesting. There are several gaps to be filled to improve this manuscript.

1. what additional information is learned that could not be determined from electrodes alone? Any insights into the mechanism of the speed, i.e. transmission type?

2. While the authors claim that it is important to combine features from imaging and from electrophysiology, they present no evidence that this combination is important for their prediction. The authors should directly compare equivalent parameters from each domain individually and determine whether the combination of features leads to better predictions. This would provide more evidence that the combination of these techniques is important.

3. It is important to show the relationship between each of the extracted features and the speed of seizures across time. Breaking each of these down by quartiles (first and last 25%) may be hiding important features about the timecourse of these parameters. In addition, it is important to examine the relationship between each of these parameters. For instance, high gamma power is likely to reflect a similar increase in activity (or excitability) that would be reflected in the calcium imaging activity measures. Investigating the relationship between these parameters would strengthen the claim that they are important to integrate together.

4. The authors need to provide additional evidence that the calcium imaging is truly recording neuronal responses and not just neuropil activation. This is particularly true for the initial wave of activation which could be driven by common inputs that are being registered as neuronal activity.

5. The authors provide minimal details about the imaging window and 4-AP delivery. Please clarify this section of the methods.

6. The data presented from the silicon probes shows high gamma and MUA relative to the low frequency component of the seizure. These plots appear to use the local LFP signature for the phase comparison. Are there differences between these LFP signatures across layers of CA1? If not, it would be pretty striking to see the MUA at different phases within each sublayer. A detailed analysis of this could be interesting and relate to the engagement of distinct sublayers of CA1 during each spike wave.

7. The rationale for examining only 3 of the 4 seizures with respect to travelling wave speed is not sufficient. The authors appear to simply dismiss 1/4 of their data simply because it doesn’t fit. This rationale needs to be clarified.

8. “we observed spikes and miniature seizures that emerge prior to seizures” - Miniature seizure is nonspecific and should be described better or a different term used, i.e. “epileptiform pattern on a single channel"

9. “Of all seizure pairs, 92% of pairs had positive correlations between cell recruitment ranks (Spearman’s r, p <0.05, 23/25 pairs, see Materials and Methods).” The authors should clarify if this is based on 23 pairs being significantly above chance or 23/25 being positive (which is itself above chance).

10. “hippocampal and cortical seizures may exhibit convergent phenomena at the scales of individual cells and neural ensembles but divergent phenomena at the network scale. Finally, drawing on the results of our data reduction and machine learning experiments, a local inhibition-dominant network process in CA1 may precipitate the transition from seizure to a post-seizure state.” Neither of these claims are particularly well supported by the data and are highly speculative. These should be moderated in the discussion

11. The paper should make a better description of how this is different from the Kuzum and Driscoll papers. Is it just the location of the device, or were different technologies used?

12. There is somewhat of a disconnect among the results described. It appears that, Figures 1 and 2 describe the model and variability of seizure onset (based on calcium). Figure 3 describes spectral phase locking at different depths during post-onset seizure discharges. Then Figure 4 describes the propagation direction and speed of early and late post-onset seizure discharges. It would be great to have an illustration defining the three analyzed phases of ictal activity (onset, post-onset early, post-onset late) and referencing these definitions in the text. The transitions between results sections could also use improvement. For example, when transitioning to the analysis of depth dependent high gamma phase coupling, it would be useful to describe how this analysis fits in to the manuscript. Is this meant to be a high temporal resolution quantification of the relationship between MEA recording and calcium imaging?

13. There seems to be a significant difference in the propagation speed measured using calcium imaging vs ephys. In figure 2 data, the recruitment duration is ∼1.2s to traverse a field of view that is ∼300uM. This translates to a speed of ∼250um/s vs what is measured in Fig 4c, ∼0.5m/s (∼2000x faster). Is this difference explained by the difference in seizure onset vs post-onset travelling wave propagation? Difference in sensitivity of calcium vs ephsy? Temporal resolution?

14. Presumably, it is not feasible to look at post-onset travelling waves in the calcium data due to the indicator being near saturation, but it would be great to compare the direction (and speed?) of seizure onset measured using the full MEA and full FOV calcium imaging. This would exploit the multi-scale nature of the multimodal data collected.

15. How were the cells in Fig 1E chosen? It appears that the 6 cells are all doing essentially the same thing, which makes it hard to appreciate the value of multicellular calcium imaging. I’m not sure what the window length 7 Savitzky-Golay filter translates to in terms of cutoff frequency, but maybe less filtering would reveal cell-to-cell differences? Substantial variability is apparent in Fig. 2B - is 1E a representative example or more homogenous than average?

16. How was 5 STD above the mean chosen as a threshold? It seams that a combination of filtering and high threshold might have the tendency to reduce onset time variability. Were other thresholds tested?

17. What statistical test was performed: “In the majority (9/12) of seizures, we found that the last 25% of discharges had a significantly lower median speed compared to the first 25% of discharges in a seizure.” It seems that it would be appropriate to perform a paired comparison of first 25% speed vs last 25% for all 12 seizures and report a p-value. It sounds like the seizures with a difference were analyzed separately, which would be a highly biased analysis.

18. Extended Fig 1-2C shows an interesting phenomenon where the calcium signal reflects a subset of the electrically recorded signal. This could be a great example of the value of calcium imaging potentially showing how neurons can combine to generate a population signal and probably warrants some added discussion.

19. If the analyses proposed above have already been done and produced negative results, they should be included as Extended data and discussed. For example, if analysis of seizure onset wave propagation using full-FOV calcium imaging data does not correlate with MEA-recorded wave propagation, it might be due to MEA electrodes picking up signal from many neurites unrelated to the imaged neurons.

20. Differences in travelling wave speed evolution near seizure termination (vs smith et al and liou et al) could also be explained by the fact that recordings were from chemically induced seizures in an otherwise non-epileptic brain. Another potentially significant difference is that the recordings were performed in a rodent brain vs human.

Minor

1. Missing word typo on page 20, “Critically, this multimodal paradigm enables us to seizure activity across these scales.”

2. Please clarify the explanation for why only 4 seizures were included in the regression tree analysis.
