# Supplementary figures and images for: Multimodal, Multiscale Insights into Hippocampal Seizures Enabled by Transparent, Graphene-Based Microelectrode Arrays
Source: eNeuro. 2022 May 9;9(3):ENEURO.0386-21.2022. doi: 10.1523/ENEURO.0386-21.2022 (PMC9087744; doi:10.1523/ENEURO.0386-21.2022)

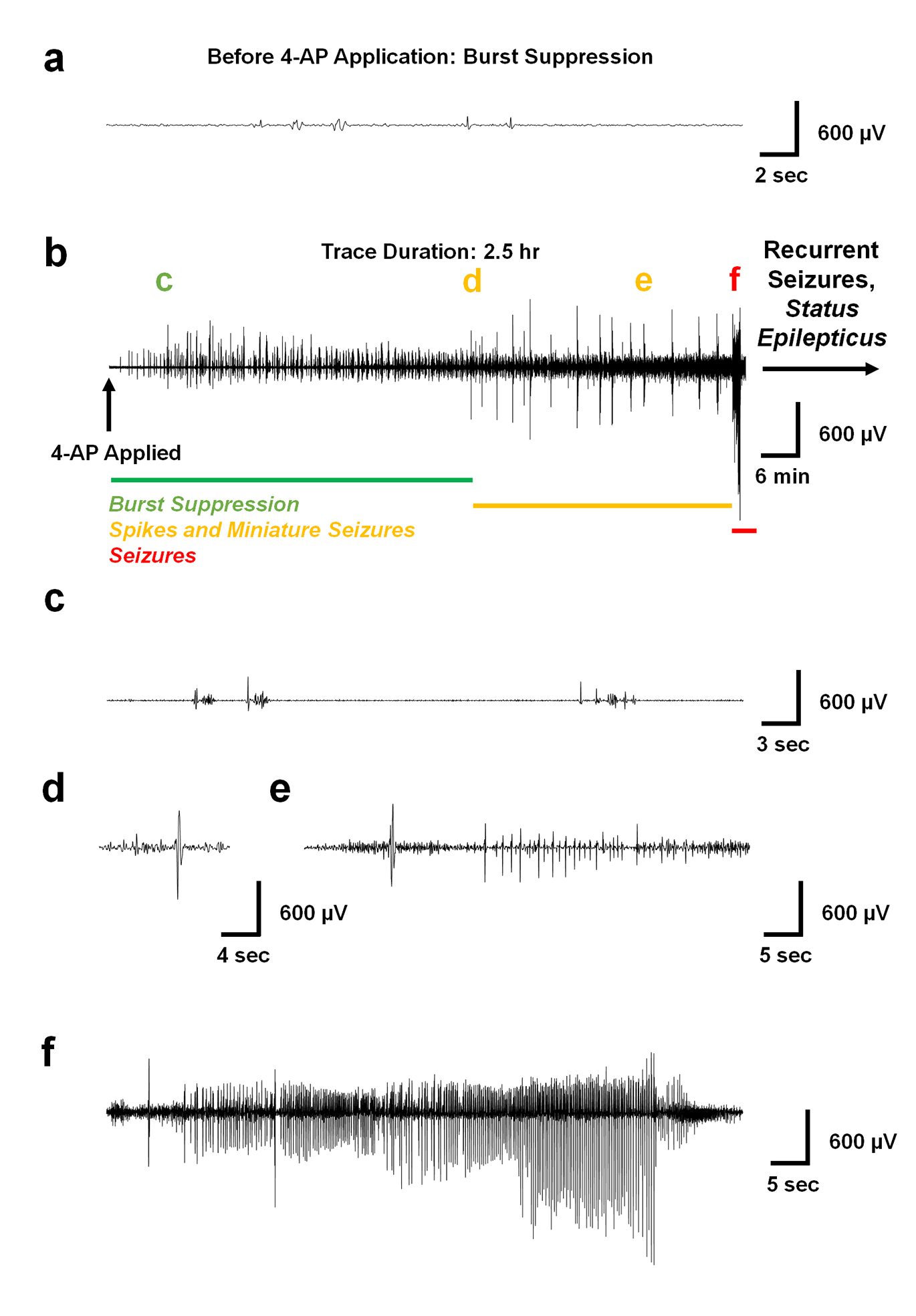

Supplement: Extended Data Figure 1-1 — Evolution of right dorsal hippocampal activity following application of 4-AP to the left cortex. In half of our experiments (1/3 Gr MEA implants, 4/7 multishank probe implants), we observed a typical evolution of epileptiform activity in the hippocampus contralateral to the site of 4-AP application to the cortical surface. All traces in Extended Data Figure 1-1 were obtained with multishank probes and from the stratum pyrimidale (sp) of CA1. A, Prior to application of 4-AP to the left cortex, the hippocampus displays low amplitude burst suppression activity. B, 2.5-h trace from sp displaying overview of the evolution of hippocampal activity following 4-AP application to the contralateral cortex. A period of burst suppression evolved to spikes and extended epileptiform patterns in 4/7 animals implanted with multishank probes. Large scale seizures later emerged in 7/7 animals implanted with multishank probes. Emergence of seizures typically was followed by multiple seizures or status epilepticus. The letters above the trace indicate the temporal locations of the activity displayed in panels C–F. C, Burst suppression activity in CA1 persists immediately after application of 4-AP to the left cortex. D, Individual spikes later emerge with a greater amplitude relative to the burst suppression activity. We defined spikes as brief, large amplitude (<1 s, >500 μV) deflections in the envelope of the electrophysiological recording. E, Spikes typically evolved into events characterized by an initial spike with relatively lower amplitude after discharges. These events could endure for up to 30 s and appeared on all recording channels. In this study, we term this type of activity an “extended epileptiform pattern.” We avoid other candidate terminologies, such as interictal polyspikes or microseizure, because the durations of polyspikes or microseizure typically do not exceed 5 s and tend to be confined to restricted areas of the brain. The progression from spikes to extended epil [file enu-eN-NWR-0386-21-s02.tif]

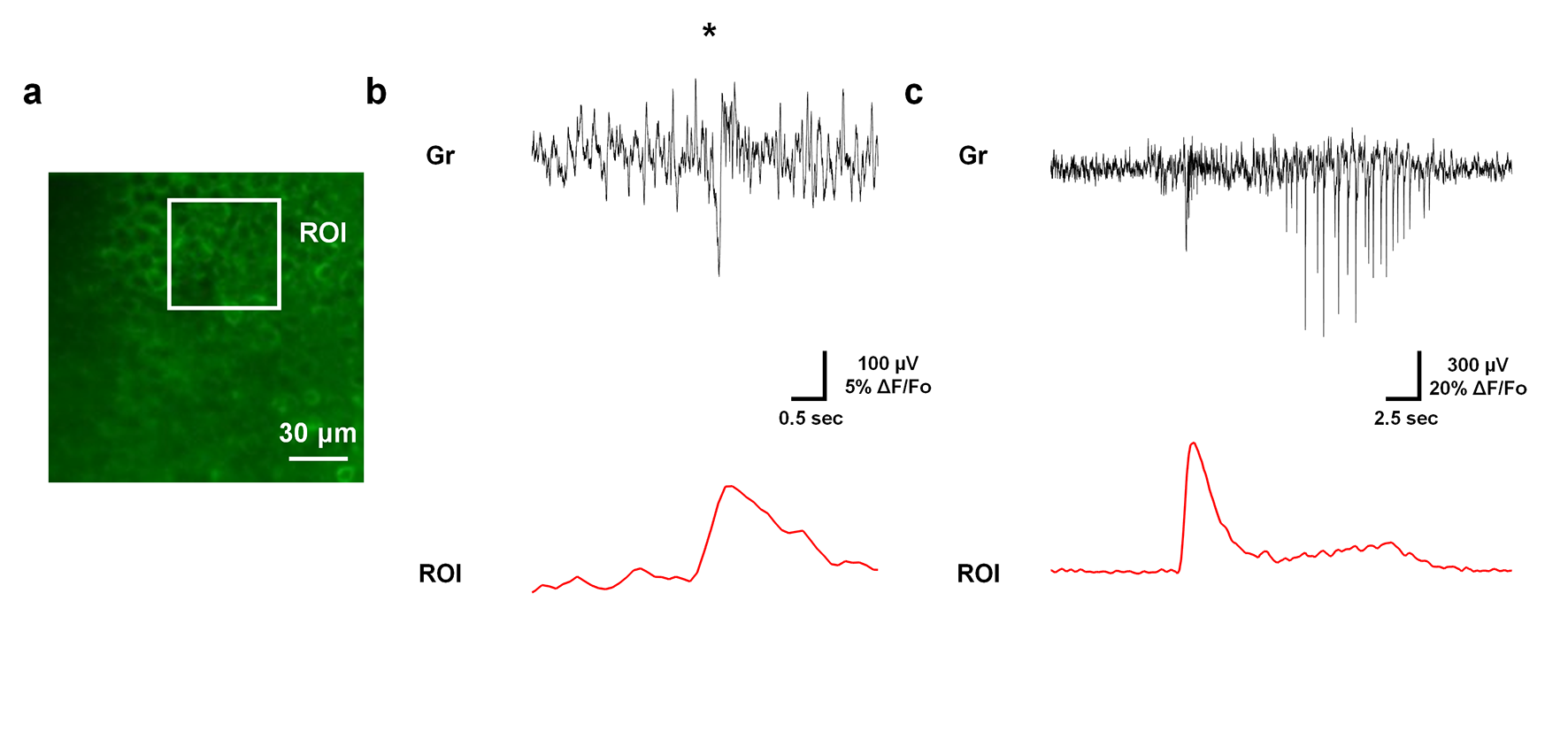

Supplement: Extended Data Figure 1-2 — Multimodal resolution of epileptiform activity enabled by Gr MEAs. Spike and extended epileptiform pattern activity were observed in 1/3 animals with high quality Gr MEA recordings. A, Representative image obtained using two-photon multicellular calcium imaging in the stratum pyrimidale of CA1. The square ROI corresponds to the approximate area covered by a single 50 × 50 μm Gr microelectrode. B, Representative spike activity, indicated with an asterisk, from a single Gr microelectrode (top) and a corresponding square ROI, indicated in panel A, from the calcium imaging (bottom). C, Representative extended epileptiform pattern activity from a single Gr microelectrode (top) and a corresponding square ROI, indicated in panel A, from the calcium imaging (bottom). Note that the calcium signal reflects a subset of the electrically recorded signal, demonstrating the potential utility of the Gr MEA to uncover how neurons may act together to generate a population signal. Download Figure 1-2, TIF file. [file enu-eN-NWR-0386-21-s03.tif]

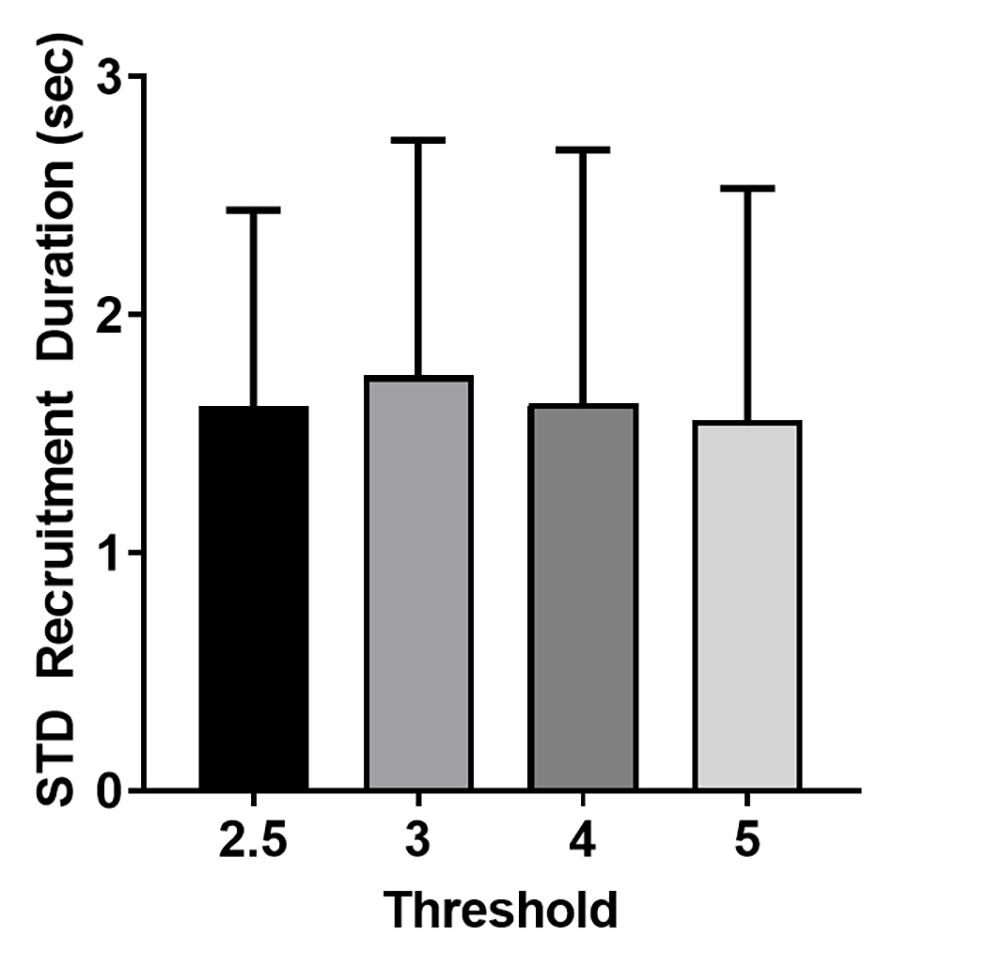

Supplement: Extended Data Figure 2-1 — Within-animal recruitment duration variability does not change with varying thresholds (ordinary one-way ANOVA, F = 0.006449, p > 0.05, dF = 3.12). Mean STD ± SEM for a threshold set at mean + 2.5, 3, 4, and 5*SD. Download Figure 2-1, TIF file. [file enu-eN-NWR-0386-21-s04.tif]

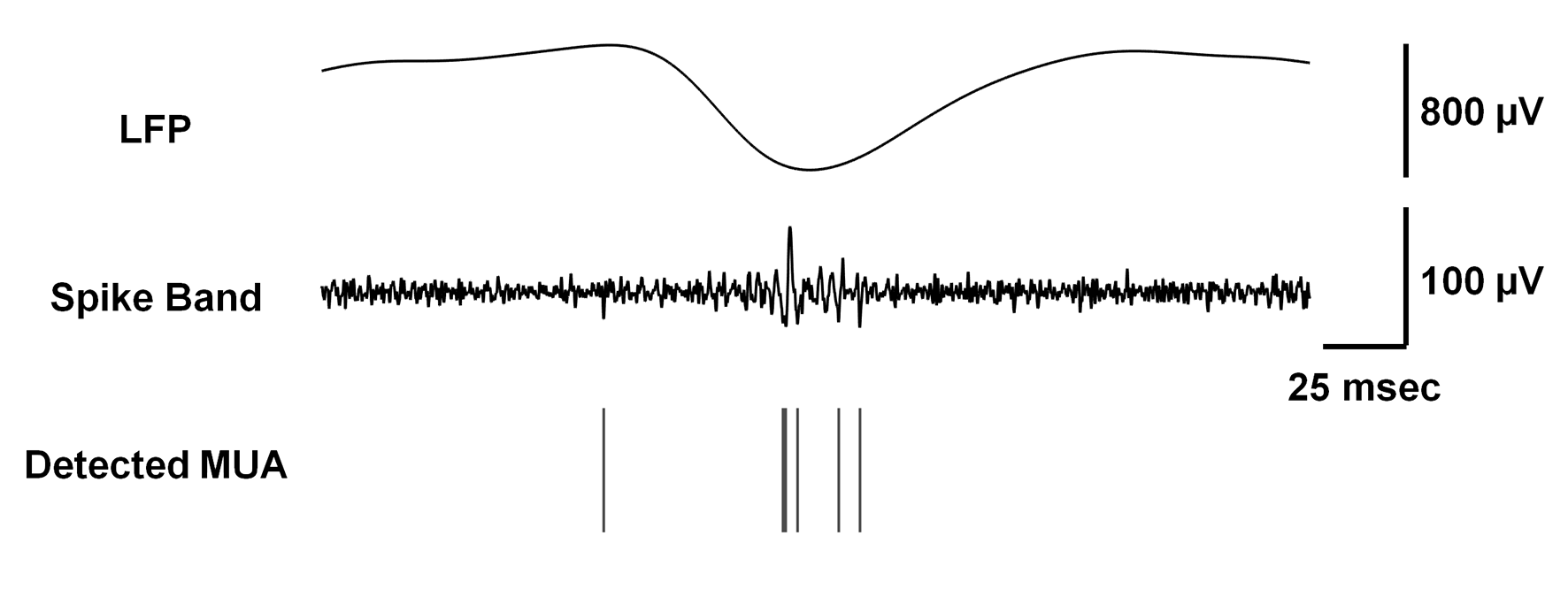

Supplement: Extended Data Figure 3-1 — Representative MUA recorded during a single seizure discharge using a Gr MEA. The traces are derived from the LFP (4–30 Hz) and the spike band (300–3000 Hz). In the bottom panel, single lines indicate time points with detected MUA. Download Figure 3-1, TIF file. [file enu-eN-NWR-0386-21-s05.tif]

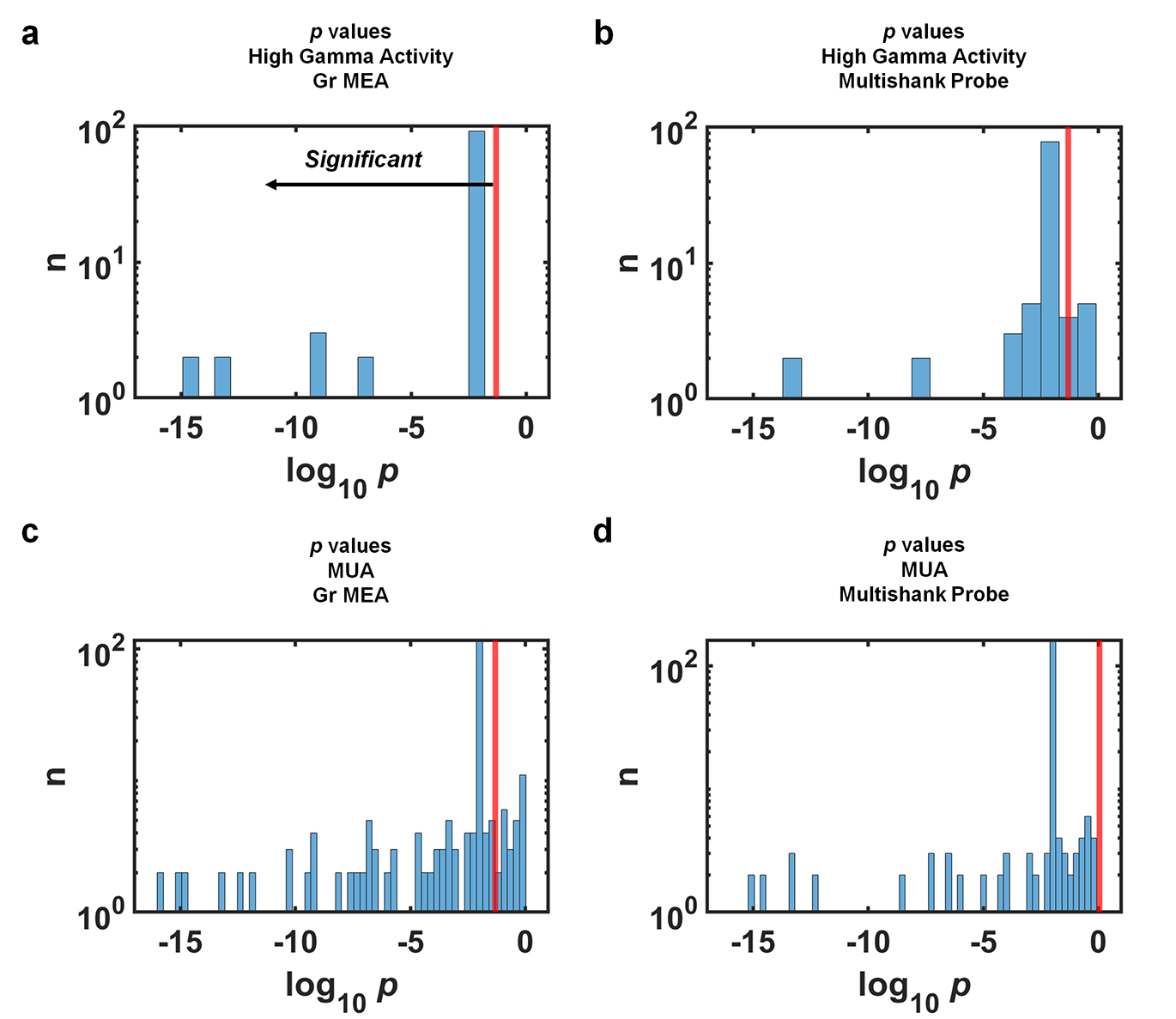

Supplement: Extended Data Figure 3-2 — Histograms of p values comparing observed high γ peak amplitude and multiunit phase distributions relative to null distributions. Note that all histograms have log10-transformed y-axes. This choice was made because all p values that were equal to 0 were manually reset to 0.01 for plotting purposes, and the samples became highly enriched at p = 0.01. All bars to the left of the red line, indicating p = 0.05, were considered significant. P values were obtained from the Watson–Williams test or the circular multisample test for equal median directions comparing the experimentally observed phase distribution and a randomly sampled phase distribution. A, Histogram of p values for comparisons of phase distributions of peak high γ amplitude versus null distributions from the Gr MEA. 100% (106/106) of channels over all seizures recorded displayed phase distributions of high γ peak amplitude that differed from null distributions. B, Histogram of p values for comparisons of phase distributions of peak high γ amplitude versus null distributions from the multishank probes. 89.2% (215/241) of channels over all seizures recorded displayed phase distributions of high γ peak amplitude that differed from null distributions. C, Histogram of p values for comparisons of phase distributions of MUA versus null distributions from the Gr MEA. 92.5% (98/106) of channels over all seizures recorded displayed phase distributions of MUA that differed from null distributions. D, Histogram of p values for comparisons of phase distributions of MUA versus null distributions from the multishank probes. 92.1% (222/241) of channels over all seizures recorded displayed phase distributions of MUA that differed from null distributions. Download Figure 3-2, TIF file. [file enu-eN-NWR-0386-21-s06.tif]

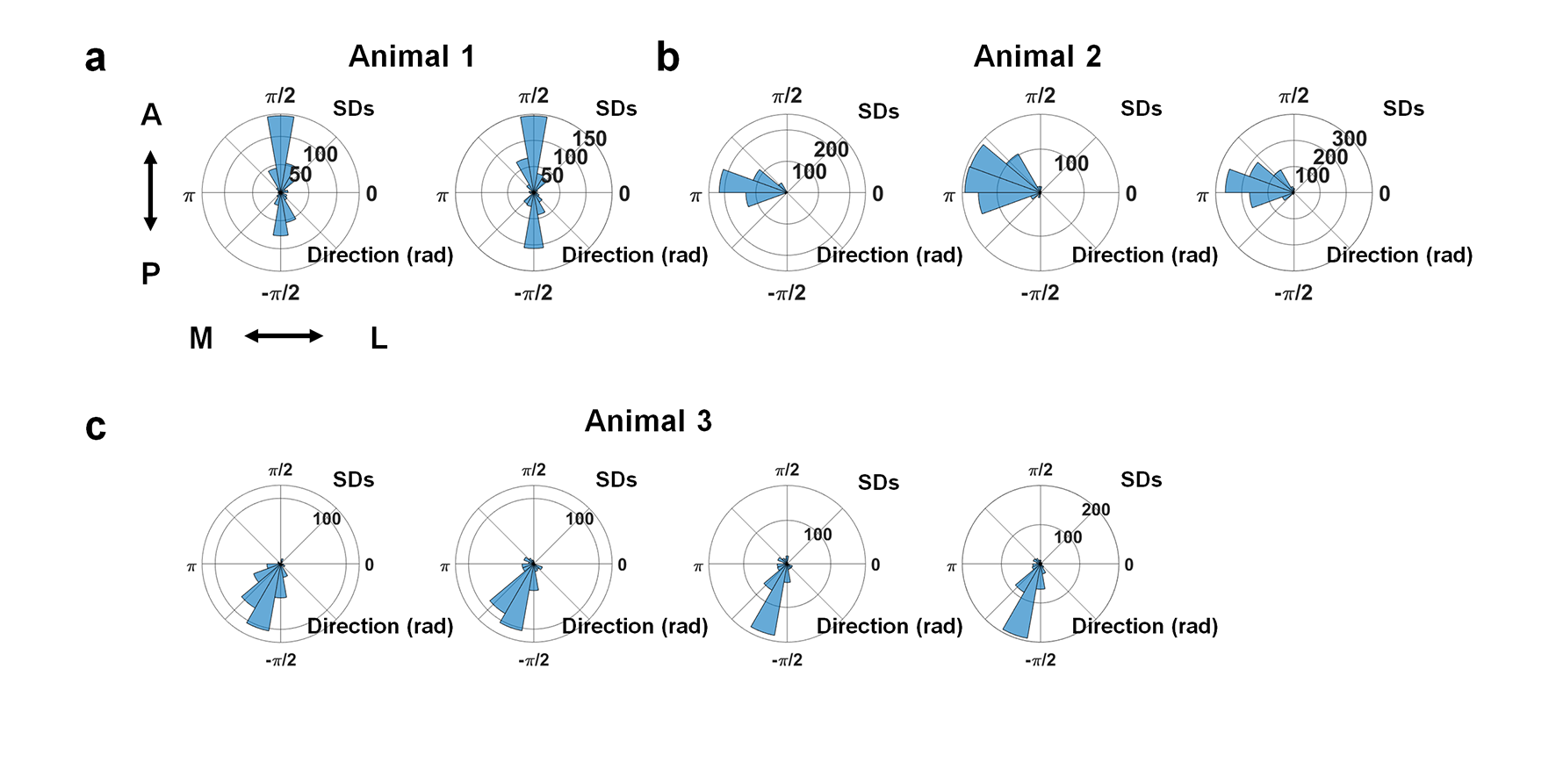

Supplement: Extended Data Figure 4-1 — Traveling wave directions of all seizures captured electrographically on the Gr MEA. All panels are oriented in the same convention as Figure 4C. As a reminder, the orientation of the angles along the A-P and M-L axes are displayed in the leftmost polar histogram of panel A. A, Distributions of the traveling wave direction for other seizures (i.e., those seizures not pictured in Fig. 4C) captures using the MEA from animal 1. B, Distributions of the traveling wave direction for other seizures captures using the MEA from animal 2. C, Distributions of the traveling wave direction for the other seizures captures using the MEA from animal 3. Download Figure 4-1, TIF file. [file enu-eN-NWR-0386-21-s07.tif]

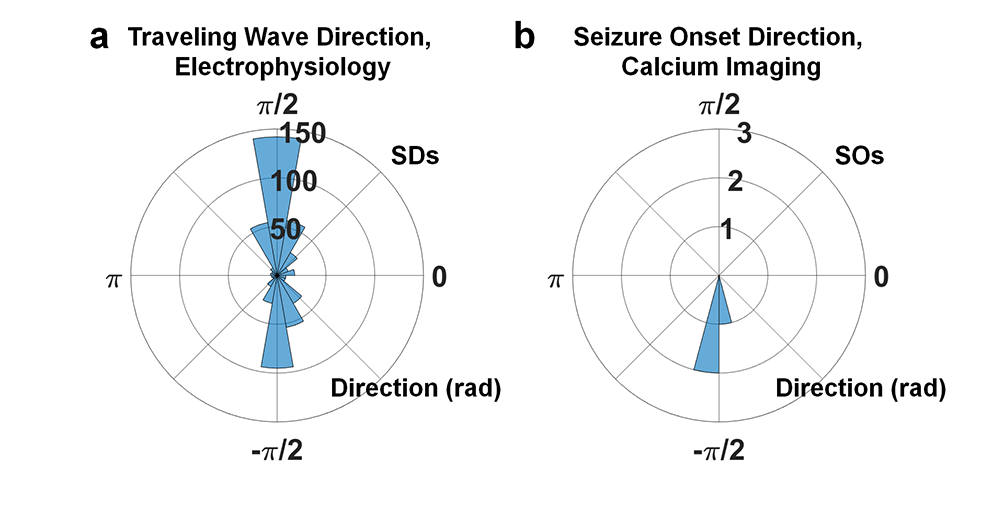

Supplement: Extended Data Figure 4-2 — Consistency of directions of electrophysiological seizure traveling waves and three seizure onsets captured via calcium imaging. Both panels are oriented in the same convention as Figure 4C and Extended Data Figure 4-1. A, Sample distribution of the traveling directions for a seizure captured using the MEA from animal 1. B, Polar histogram showing the distribution of the directions of seizure onsets (SOs) captured using multicellular calcium imaging. Comparisons of traveling wave speeds between modalities are not included due to the relatively slow calcium imaging sampling frequencies of 5 Hz used in this study. Download Figure 4-2, TIF file. [file enu-eN-NWR-0386-21-s08.tif]

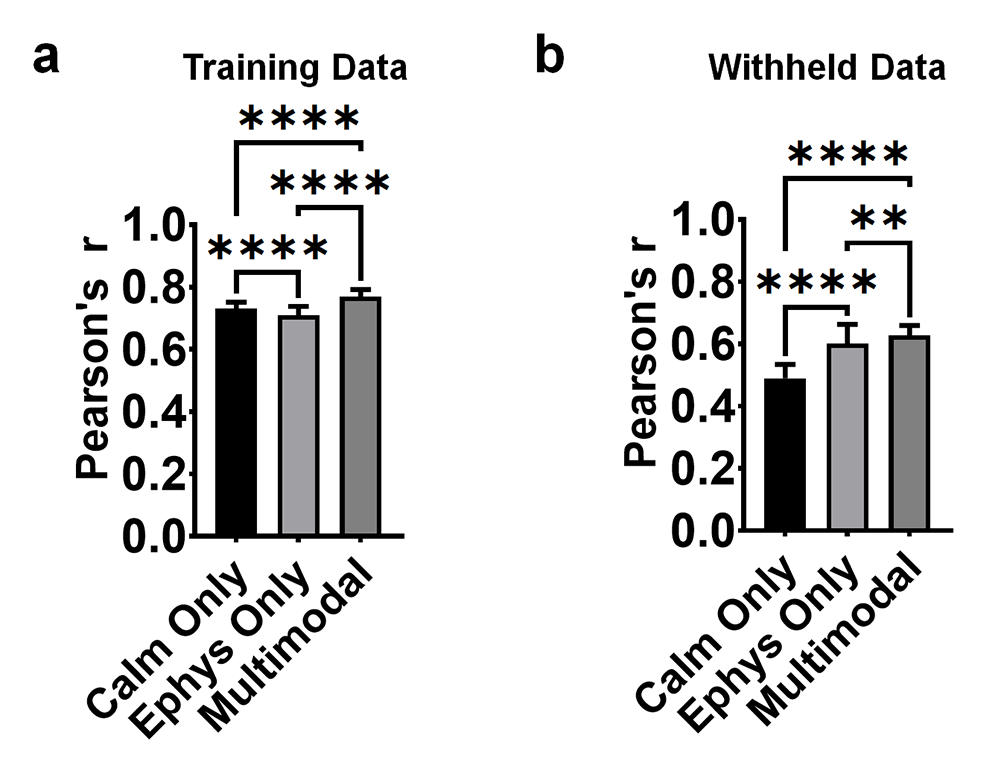

Supplement: Extended Data Figure 5-2 — Regression trees trained on multimodal features outperform trees trained on features derived from single-modal features. A, Performance of models trained on single modal and multimodal features to predict traveling wave speeds in the training dataset. The performance was measured as the Pearson’s r between the true and predicted traveling wave values. Here, the mean Pearson’s r over 1000 model predictions for the three different model types is shown, and the error bar represents the SD. The three groups all have medians that differ from each other (Kruskal–Wallis test, K-W statistic = 1640, p < 0.0001, Dunn’s multiple comparisons tests, p < 0.0001 for all). B, Performance of top 100 best-performing models trained on single modal and multimodal features to predict traveling wave speeds in a dataset withheld from model training. Here, the mean Pearson’s r over 100 model predictions for the three different model types is shown, and the error bar represents the SD. The three groups all have medians that differ from each other (Kruskal–Wallis test, K-W statistic = 180.4, p < 0.0001, Dunn’s multiple comparisons tests, p < 0.01 for all). Download Figure 5-2, TIF file. [file enu-eN-NWR-0386-21-s10.tif]

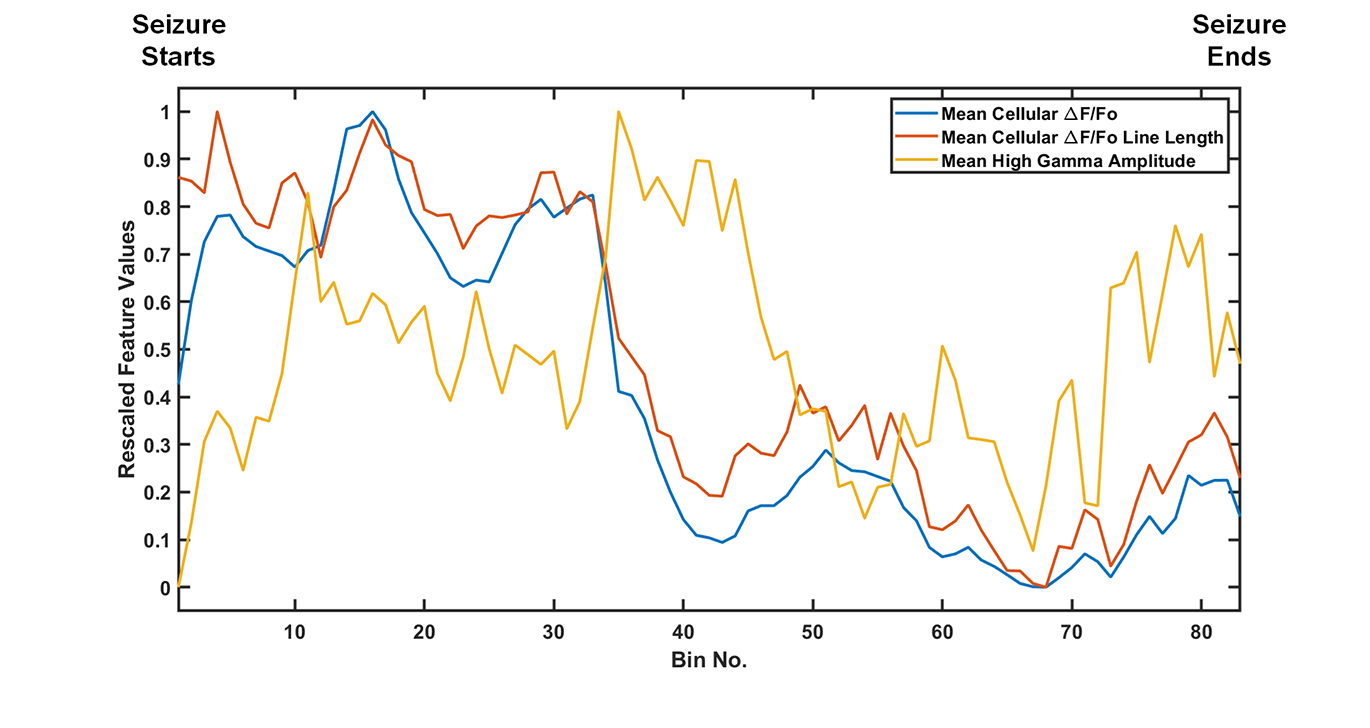

Supplement: Extended Data Figure 5-3 — Representative time evolution of rescaled values of the three most important features for one seizure. The entire seizure duration shown is 330 s. Download Figure 5-3, TIF file. [file enu-eN-NWR-0386-21-s11.tif]

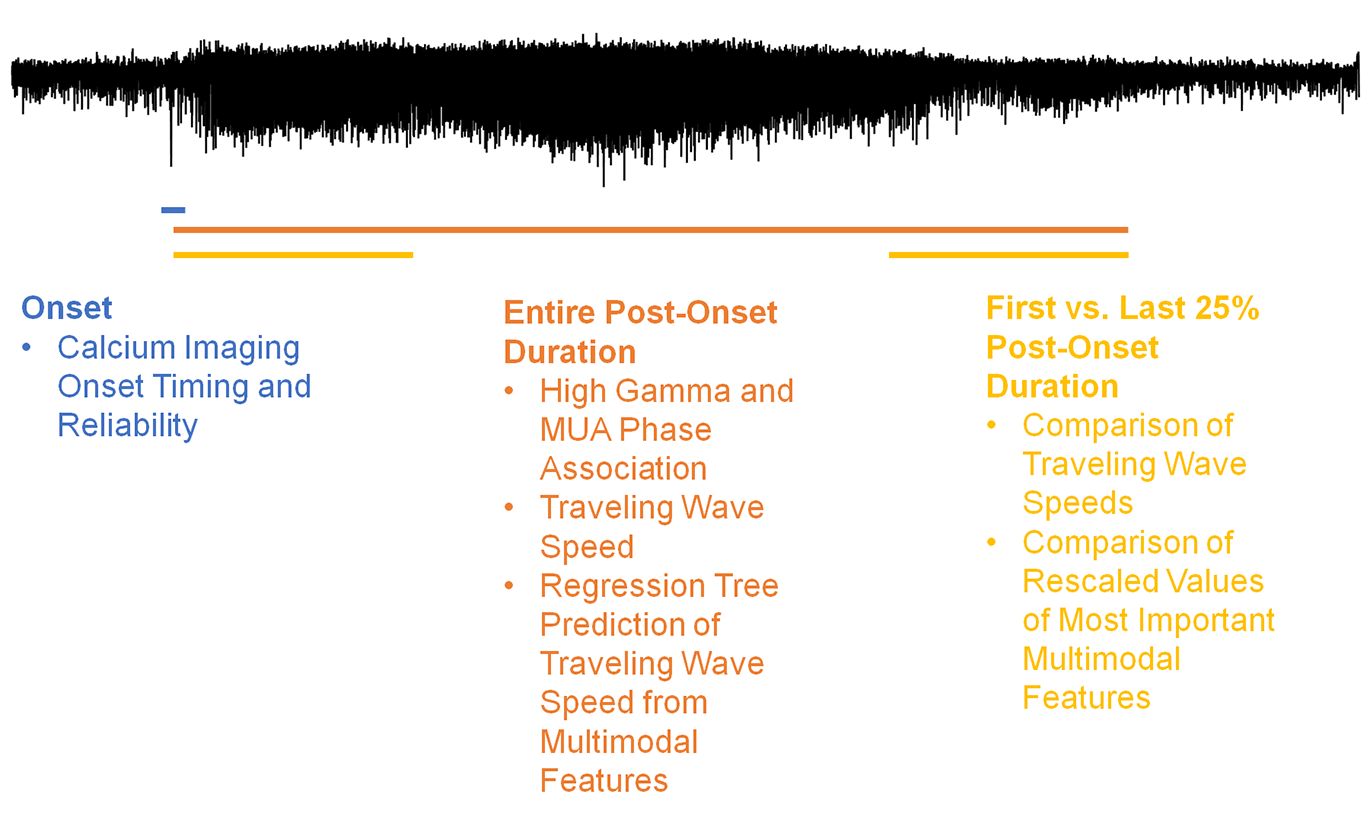

Supplement: Extended Data Figure 6-1 — Summary of analyses presented in this work relative to seizure evolution. Download Figure 6-1, TIF file. [file enu-eN-NWR-0386-21-s12.tif]
