# Supplementary material for: Multimodal, Multiscale Insights into Hippocampal Seizures Enabled by Transparent, Graphene-Based Microelectrode Arrays
Source: eNeuro. 2022 May 9;9(3):ENEURO.0386-21.2022. doi: 10.1523/ENEURO.0386-21.2022 (PMC9087744; doi:10.1523/ENEURO.0386-21.2022)
Supplement: Extended Data Figure 5-1 — Feature importance values in 1000 bagged regression trees and the top 10% generalizing models. The three most important features are indicated in bold. Download Figure 5-1, DOC file. [file enu-eN-NWR-0386-21-s09.doc]

**Extended Data Figure 5-1**

|  | **Median Feature Importance, All 1000 Models** | **Median Feature Importance, Top 10% Generalizing Models** |
| --- | --- | --- |
| **Mean HG Amplitude** | 0.7496 | 0.7915 |
| Standard Deviation of HG Amplitude | 0.5871 | 0.6014 |
| Fractional Low Frequency Power | 0.5321 | 0.5354 |
| Fractional HG Power | 0.6924 | 0.6849 |
| PLV | 0.5565 | 0.6117 |
| **Mean ΔF/Fo Amp.** | 0.9040 | 0.8918 |
| **Mean ΔF/Fo L.L.** | 0.8789 | 0.8149 |
| Standard Deviation of d/dt ΔF/Fo | 0.6932 | 0.6719 |
| Mean Cell Pair Correlation | 0.3520 | 0.3545 |
| Standard Deviation of Cell Pair Correlations | 0.3859 | 0.3952 |
